# Inflammatory cytokine production in tumor cells upon chemotherapy drug exposure or upon selection for drug resistance

**DOI:** 10.1371/journal.pone.0183662

**Published:** 2017-09-15

**Authors:** Derek W. Edwardson, Justin Boudreau, Jonathan Mapletoft, Carita Lanner, A. Thomas Kovala, Amadeo M. Parissenti

**Affiliations:** 1 Ph.D. Program in Biomolecular Science, Laurentian University, Sudbury, Ontario, Canada; 2 Department of Biology, Laurentian University, Sudbury, Ontario, Canada; 3 Department of Chemistry and Biochemistry, Laurentian University, Sudbury, Ontario, Canada; 4 Division of Medical Sciences, Northern Ontario School of Medicine, Sudbury, Ontario, Canada; 5 Health Sciences North Research Institute, Sudbury, Ontario, Canada; 6 Faculty of Medicine, Division of Oncology, University of Ottawa, Ottawa, Ontario, Canada; University of South Alabama Mitchell Cancer Institute, UNITED STATES

## Abstract

Tumor Necrosis Factor alpha (TNF-α) has been shown to be released by tumor cells in response to docetaxel, and lipopolysaccharides (LPS), the latter through activation of toll-like receptor 4 (TLR4). However, it is unclear whether the former involves TLR4 receptor activation through direct binding of the drug to TLR4 at the cell surface. The current study was intended to better understand drug-induced TNF-α production in tumor cells, whether from short-term drug exposure or in cells selected for drug resistance. ELISAs were employed to measure cytokine release from breast and ovarian tumor cells in response to several structurally distinct chemotherapy agents and/or TLR4 agonists or antagonists. Drug uptake and drug sensitivity studies were also performed. We observed that several drugs induced TNF-αrelease from multiple tumor cell lines. Docetaxel-induced cytokine production was distinct from that of LPS in both MyD88-positive (MCF-7) and MyD88-deficient (A2780) cells. The acquisition of docetaxel resistance was accompanied by increased constitutive production of TNF-αand CXCL1, which waned at higher levels of resistance. In docetaxel-resistant MCF-7 and A2780 cell lines, the production of TNF-α could not be significantly augmented by docetaxel without the inhibition of P-gp, a transporter protein that promotes drug efflux from tumor cells. Pretreatment of tumor cells with LPS sensitized MyD88-positive cells (but not MyD88-deficient) to docetaxel cytotoxicity in both drug-naive and drug-resistant cells. Our findings suggest that taxane-induced inflammatory cytokine production from tumor cells depends on the duration of exposure, requires cellular drug-accumulation, and is distinct from the LPS response seen in breast tumor cells. Also, stimulation of the LPS-induced pathway may be an attractive target for treatment of drug-resistant disease.

## Introduction

Breast cancer has a mortality rate second only to lung cancer [1,2]. Surgery is the primary treatment for most breast tumors in North America, followed by radiation and/or systemic adjuvant chemotherapy [3]. Neoadjuvant or 'preoperative' chemotherapy is more common in other jurisdictions (namely Europe) and is often used worldwide to shrink tumors that are initially inoperable, permitting better surgical margins [4], as with locally advanced or inflammatory forms of the disease [5,6]. Despite continuous improvements in the treatment of solid tumors, response rates to chemotherapy are still relatively low and treatment side effects can be quite debilitating for patients. Treatment regimens for breast cancer in an adjuvant or neoadjuvant setting typically contain an anthracycline (doxorubicin or epirubicin) and a taxane (paclitaxel or docetaxel) [7]. The taxanes interact with β-tubulin, blocking the depolymerization of microtubules, and inhibiting cell division during mitosis [8,9]. As with breast cancer, treatment of ovarian cancer typically involves surgical removal of the tumor followed by adjuvant chemotherapy. Preoperative chemotherapy followed by interval debulking is also used in certain cases of advanced ovarian cancer [10]. In both instances, the chemotherapy drugs used typically involve the taxanes and a platinating agent [11].

Although the above chemotherapy agents have been shown to inhibit breast or ovarian tumor growth directly, *in vitro*, considerable evidence suggests that therapeutic benefit during treatment likely relies on an immunoadjuvant effect stemming from tumor cell death by different chemotherapy agents [12]. This effect involves the release of signaling molecules from tumor cells, serving as a trigger for a host immune response against the tumor. Various members of the family of damage-associated molecular patterns (DAMPs) have been shown to play a role as immunoadjuvants [13] emanating from dying tumor cells [14]; however, the full breadth of tumor-derived signals that are provoked during chemotherapy and that play a role in promoting a chemotherapy-induced tumor-targeted immune response is not clear.

TNF-α is an inflammatory cytokine considered to be a master regulator of innate and adaptive immune responses [15,16] and a key player in many inflammatory disorders [17]. It has been shown to be released by and impart effects on a variety of cell types, including myeloid cells [18–20], endothelial [21], epithelial cells [22], as well as tumor cells. Recently, it has been shown in *vitro* that TNF-α is released by breast and ovarian tumor cells in response to taxane exposure [23]. The release of soluble factors, such as TNF-α, from tumor cells may be of importance in chemotherapy response, with and without the involvement of the host immune system. Cancer patients may not receive a benefit from chemotherapy due to innate resistance to chemotherapy drugs, involving pre-existing tumor characteristics, or due to acquired resistance, involving changes within the tumor or its microenvironment during treatment. The presence of a variety of factors including inflammatory cytokines [TNF-α, CXCL8 (interleukin-8), and CXCL1 (GRO-α)] have been implicated in mediating both innate and acquired resistance to taxanes and/or platinating agents in tumor cell lines [23–26]. Furthermore, the production of TNF-α by malignant cells in mice has been shown to affect tumor-associated myeloid cell activity, in turn affecting tumor growth [19]. TNF-α can also stimulate cell death pathways in tumors, as docetaxel-induced TNF-α production was shown to be cytotoxic in breast tumor cells via autocrine signaling [23]. It can also affect the tumor vasculature [27], which is an important element in the treatment of solid tumors. Poor treatment efficacy can be the result of inadequate drug exposure or penetration of the tumor, both of which can be due to a variety of factors including changes or anomalies in vascular architecture [28].

Chemotherapy-induced cytokine release has been reproducibly observed in mouse myeloid cells and it is thought to be mediated by activation of the pathogen recognition receptor known as toll-like receptor 4 (TLR4) [29,30]. However, the mechanism of chemotherapy-induced cytokine release in tumor cells is less understood. We hypothesize (a) that TNF-α release can be induced by a variety of chemotherapy agents, (b) that docetaxel-induced TNF-α release is an active cellular process, and (c) that the primary mechanism of *docetaxel-induced* TNF-α release is *not* attributable to *direct* ligand-binding of drug to TLR4 at the surface of human tumor cells. Multiple groups hold the view that taxanes activate TLR4 directly as a ligand in tumor cells [30–34] despite a lack of definitive evidence in tumor cells. We show evidence in human tumor cells that docetaxel-induced TNF-αrelease requires intracellular accumulation of drug. We also show that the molecular pathway involved in docetaxel-induced TNF-α release is distinct from that of lipopolysaccharides (LPS). Moreover, it does *not* require the presence of the TLR4 adaptor protein MyD88. We also show that the acquisition of resistance to docetaxel is accompanied by distinct changes in the expression of a variety of inflammatory cytokines, including TNF-α, CXCL1, and CXCL8. Finally, we demonstrate that stimulation of the TLR4 pathway *in vitro* can increase the sensitivity of tumor cells to the chemotherapy agent docetaxel, thus identifying a possible approach to increase the efficacy of this drug in docetaxel-resistant disease.

## Methods

### Cell culture and cell lines

MCF-7 cells were obtained from the American Tissue Culture Collection, while A2780 cells were from the European Collection of Cell Cultures. Cells were cultured in DMEM and RPMI media using Sarstedt and Corning T75 tissue culture flasks for MCF-7 and A2780 cells, respectively, in a 5% CO_2_ atmosphere at 37°C. Culture media was supplemented with 10% FBS (v/v) and 1% Penicillin and Streptomycin (v/v). At confluence, cells were washed with PBS, treated with 0.25% trypsin for resuspension, after which 1 ml of complete media was added to inhibit trypsin activity. Cell suspensions were harvested by centrifugation at 650 x g for 7 minutes and resuspended in medium for counting. Docetaxel-resistant variants were generated from MCF-7 cells and A2780 cells as previously described by Guo et al. [35] and Armstrong et al. [36], respectively. MCF-7_TXT7_, MCF-7_TXT8_, MCF-7_TXT9_, MCF-7_TXT10_, MCF-7_TXT11_, and MCF-7_TXT12_ cells are MCF-7 cell lines selected for survival in progressively increasing concentrations (doses) of docetaxel [also known as Taxotere® (TXT)]. These doses (7 through 12) are 0.37, 1.1, 3.3, 5, 15, and 45 nM docetaxel, respectively. Likewise, A2780_DXL10_, A2780_DXL11_, and A2780_DXL12_ cells represent a series of A2780 cell lines selected for survival in up to 1.97 nM (dose 10), 2.96 nM (dose 11), and 8.88 nM (dose 12) docetaxel, respectively. During the above selections for docetaxel resistance, equivalent cultures of MCF-7 and A2780 cells were passaged in the absence of drug to control for differences in cell properties and behavior due to increased passage number. One of these “co-cultured control” cell lines at selection dose 10 for the MCF-7 cell line and at selection dose 12 for the A2780 cell line (MCF-7_CC10_ and A2780_CC12_ cells, respectively) served as the drug-sensitive control cell line in experiments.

### ELISA (enzyme-linked immunosorbent assay)

All ELISAs were completed using kits from R&D systems (Minneapolis, MN, USA), following the manufacturer’s experimental protocol. Plates pre-coated with monoclonal antibody were purchased for the detection of the cytokines TNF-α, CXCL8, and CXCL1 (catalog numbers DTA00C, D8000C and DGR00, respectively). Equal numbers of cells were transferred to 10 cm culture plates and allowed to adhere overnight. After treatment with docetaxel in the absence or presence of various additional agents for up to 96 hours, the culture media were collected and subjected to centrifugation at 875 x g to remove cell debris. The media supernatants were then transferred to a 50 ml Amicon tube equipped with a 3 kDa molecular-weight-cut-off filter (EMD Millipore, Etobicoke, ON; cat no. UFC900324) and subjected to centrifugation at 3273 x g. The volumes of media supernatants were measured and the samples stored at -80°C. ELISA standards were prepared by serial dilution of a reconstituted lyophilized stock solution. After 2 hours of incubation at room temperature, the wells were rinsed with wash buffer. A horseradish peroxidase (HRP)-conjugated secondary antibody was then added to each well and the plate incubated for 1 hour at room temperature, followed by a rinse with wash buffer and addition of HRP-sensitive colorimetric substrate for 20 minutes. The absorbance of the samples at 450 nm and 540 nm was measured using a Synergy H4 Hybrid spectrophotometer from BioTek® and duplicate measurements were averaged for both samples and standards. After subtracting the absorbance at 540 nm from the absorbance at 450 nm absorbance values were likewise corrected for background absorbance of standard diluent (for standards) or concentrated tissue culture medium (for samples).

### Cell counting and trypan blue staining

Following trypsinization and staining with 0.066% trypan blue (v/v), cells were counted in a hemocytometer (Improved Neubauer, Hausser Scientific) using a light microscope (Leica - 10x/0.22) with phase contrast filter. The average number of trypan-positive and trypan-negative cells within a 4x4 grid of 4 different fields was determined.

### Reverse-transcription of mRNAs and quantitative PCR (polymerase chain reaction)

Two million cells were transferred to 10 cm tissue culture dishes and grown for 30, 36, and 42 hours. RNA was isolated using a Quiagen RNeasy extraction kit (Qiagen, Inc., Toronto, ON). The cells were washed with PBS, and treated with 350 μl of RLT buffer containing 3.5 μl of β-mercaptoethanol. Cells were harvested using a cell-scraper, and the lysate was subjected to 4–5 passes through a 16-gauge needle. After transfer of the lysate to a microfuge tube, an equal volume of 70% ethanol was added, and after mixing, the mixture was transferred to a Qiagen mini-column and RNA purified as described in the manufacturer’s protocol. The concentration of purified RNA was determined by measuring the absorbance at 260 nm in a NanoDrop 2000C spectrophotometer (Thermo Scientific), with a baseline correction for absorbance at 340 nm. Two μg of purified RNA was diluted to generate a mixture of 15 μl, containing 2 μl of 10x DNase buffer, and 2 ul of DNase (1 unit/μl). After a 15 minute incubation at room temperature, 2 μl of 25 mM ethylenediaminetetraacetic acid (EDTA), 20 μl of T20 primers (20 ng/μl) and 16 μl of dNTP’s (10 mM) were added. Samples were incubated for 5 minutes at 65°C, after which 16 μl of 5x first-strand buffer and 8 μl of dithiothreitol (DTT) were added, with a subsequent incubation period of 2 minutes at 37°C. Finally, 200 U of Moloney-murine leukemia virus (M-MLV) reverse-transcriptase was added and the mixture left for 2 hours at 37°C to allow for reverse-transcription of mRNA to cDNA. Samples were then heated for 5 minutes at 95 ^o^C to inactivate the transcriptase.

Quantitative real-time PCR reactions were performed in a 96-well plate using cDNA preparations. mRNA standards were generated using cDNA from an untreated sample originating from the same cell line as the other samples, to generate a standard curve. Five or more 2-fold serial dilutions were made starting with an initial 4-fold dilution in RNase-free water. Samples were prepared beginning with a 16-fold dilution (3 μl of sample added to 45 μl of RNase-free water). The primer pair for detection of TNF-α transcripts was 5'-CCT GCC CCA ATC CCT TTA TT-3' (forward) and 5'-CCC TAA GCC CCC AAT TCT CT-3' (reverse). A primer pair with sequences 5'-TCC ATC ATC CGC AAT GTA AAA-3' (forward) and 5'GCT TCT CGC TCT GAC TCC AAA-3' (reverse) was also used to quantify expression of the S28 reference gene. All primers were purchased from Integrated DNA Technologies (Skokie, IL, United States). Triplicate samples were prepared for qPCR with 5 μl per well of cDNA standards, including a no-template control containing only RNase-free water, or 5 μl per well of cDNA for unknown samples, to which was added 7.5 μl of 2 mM pooled forward and reverse primers and 12.5 μl per well of 1x SYBR Green. The plate was centrifuged for 1 minute at 650 x g in order to position all reagents at the bottom of each well. qPCR involved a hot start for 5 minutes at 95°C, followed by 40 cycles consisting of: 30 seconds at 95°C, 30 seconds at 53°C, and 1 minute at 72°C.

### Flow cytometry

Cells (2 x 10^5^ per well) were allowed to adhere overnight in 6-well plates. The cells were rinsed with PBS, resuspended with 500 μl of 0.25% trypsin, transferred to 1 ml microfuge tubes, combined with 500 μl of PBS, and centrifuged for 3 minutes at 300 x g. The pelleted cells were resuspended in a mixture of 20 μl of phycoerythrin-conjugated monoclonal antibody, specific for P-glycoprotein (P-gp), protected from light and left at room temperature for 30 minutes. Cells were centrifuged for 3 minutes at 300 x g, washed with PBS, resuspended in PBS and then analyzed with an FC500 flow cytometer (Beckman Coulter, Mississauga, ON) set to measure fluorescence using the FL2 filter for 10,000 events per sample with no gating. Mean fluorescence Intensity (MFI) values for the mouse anti-human P-gp antibody or for an IgG2β isotype control (BD Bioscience, catalog 557003 and 555743, respectively) were recorded and the MFI for the isotype control subtracted from the MFI for the P-gp-specific antibody to correct for non-specific binding.

### Drug uptake measurements

Cells (2x10^5^) were plated in 6-well plates and left to adhere overnight in the appropriate medium. Cells were then incubated with either 2.5 nM tritium-labeled docetaxel (^3^H-TXT from American Radiolabeled Chemical, St. Louis, MO) or 100 nM Tariquidar (Med Chem Express, Monmouth Junction, NJ) or a combination thereof, with 5% CO_2_ at 37°C. After 12 hours, the media were removed, while the adhered cells were rinsed with 1 ml PBS, treated with 0.25% trypsin, and then placed in 5 ml of scintillation fluid. The radioactivity associated with the cells was then quantified using a Beckman LS 6000 IC scintillation counter.

### Cell protein extraction

Two million A2780 and MCF-7 cells were seeded onto 10 cm tissue culture plates and cultured for 24 and 48 hours, respectively. Protein extracts were prepared from these cells by lysis in 500 μl of chilled RIPA buffer [1% NP-40 (Sigma, St. Louis, MO), 0.5% sodium deoxycholic acid (Sigma, Oakville, ON) and 1% SDS (BioRad, Mississauga, ON) in PBS] supplemented with 2 mM sodium orthovanadate (Sigma) and 1X protease inhibitor cocktail (Roche, Mississauga, ON). Cell lysates were passed through a 21-gauge needle 5 times, incubated for 30 minutes on ice, and then centrifuged at 15,000 x g for 30 min. The supernatants were divided into aliquots that were stored at -80°C. The protein concentration of extracts was quantified using the Pierce BCA protein assay kit (Thermo-Fisher, Mississauga, ON). Alternatively, cells were extracted in 5X SDS-PAGE sample buffer to ensure complete dissolution of membranes for enhanced isolation of membrane-associated proteins.

### Immunoblotting experiments

Lysates (36 μg of protein) were loaded onto a 10% polyacrylamide gel for electrophoresis, transferred to a nitrocellulose membrane using a BioRad Trans-blot® SD Semi-Dry Transfer Cell for 1hr at 12V. The membrane was blocked in 5% skim milk in TBST [0.24% Trizma® Base (Sigma), 0.8% NaCl (Fisher), 0.1% Tween20® (Sigma) at pH 7.6] for 1 hour before incubation overnight at 4°C with a human TLR4 antibody (1:250, Santa Cruz, Dallas, TX), human MyD88 antibody (1:1000, Cell Signaling, Danvers, MA), human TRIF antibody (1:700, Cell Signaling, Danvers, MA) or a GAPDH antibody (1:10,000, Santa Cruz). All primary antibodies were diluted in TBST supplemented with 5% BSA (Sigma). Membranes were washed with TBST 3 times for 5 minutes. TLR4 and MyD88 levels were detected using an HRP-conjugated goat anti-rabbit secondary antibody (1:10,000, Santa Cruz). The membranes were washed before incubation with ECL reagent (Santa Cruz) and exposure on CURIX ortho HTL-Plus film (AGFA Healthcare, Waterloo, ON). Densitometry was performed using the FluorChem FC3 apparatus and AlphaEaseFC 4.0 software.

### Clonogenic assays

Cells were added to 6-well plates (3x10^5^ cells) with 2 ml of media per well and left to adhere overnight. Each well received one of twelve different concentrations of docetaxel (including a ‘no drug’ control) and the cells incubated for 24 hours. After 24 hours the medium in each well was collected, while the adherent cells were resuspended using 0.25% trypsin. The floating and adherent cells from each well were combined, centrifuged at 650 x g for 7 minutes, and resuspended in 300 μl of culture media. The cells were then added to 2.7 ml of methylcellulose (with 25% FBS v/v), and mixed thoroughly by vortexing. After incubating an hour to allow bubbles to escape, 1.2 ml of each cell suspension were transferred to a 6-well plate. The plates were then incubated for two weeks and photographs of 12 independently chosen fields were taken using a Leica light microscope with a Leica 4x/.10 objective lens. Viable colonies, defined as being greater than or equal to 4 mm (MCF-7) or 3 mm (A2780) in two perpendicular directions (as measured on a PowerPoint slide and printed 4 slides per 8'x11' page), were counted. The average colony count was then computed and divided by the average colony count for the untreated control to obtain the survival fraction. Survival fractions were then plotted against drug dose and a sigmoidal dose-response (survival) curve was fit to 12 data points using GraphPad Prism software. The concentration that inhibited colony formation by 50% (IC_50_) was determined for each dataset at the inflection point of the sigmoidal curve. Clonogenic assays using Tariquidar involved 24-hour co-administration of Tariquidar and docetaxel together, whereas clonogenic assays with LPS involved a 48-hour pretreatment with LPS followed by a 24-hour docetaxel treatment. Tariquidar clonogenics used 3 x 10^5^ MCF-7 cells and 2 x 10^5^ A2780 cells, whereas LPS clonogenics used 2 x 10^5^ and 1 x 10^5^ cells for MCF-7 and A2780 cells, respectively.

### Statistical analyses

Statistical analyses are specified in figure legends for each experiment. They involved comparison of the mean values from data obtained from at least three replicates (separate cell culture dishes). Two-tailed T-tests were performed when comparing data, from cell populations, with one independent variable (ie. drug concentration or time after treatment). 2-way Analysis of Variance (ANOVA) was performed for experiments comparing data from cell populations with two independent variables (ie. both drug concentration and time).

## Results

### Characterization of docetaxel-induced TNF-α release

#### Assessing the effects of drug concentration and exposure time on TNF-α release

**Fig 1:**
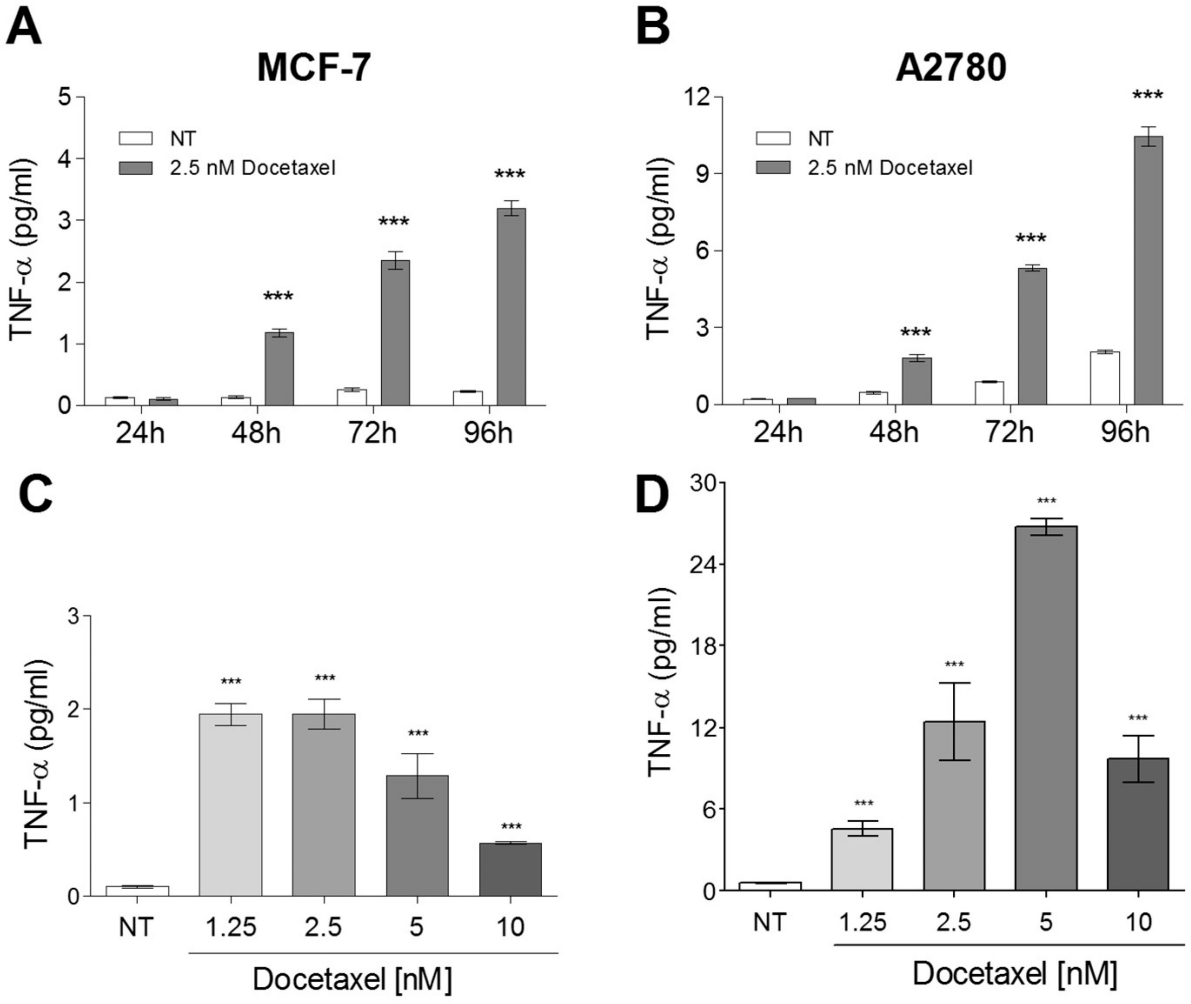
Docetaxel-induced TNF-α levels in media of breast and ovarian tumor cells are dose- and time-dependent. A) and B) Effects of treatment time on TNF-α release by 2.5 nM docetaxel from MCF-7 and A2780 cells; C) and D) Effects of docetaxel concentration on TNF-α release in breast and ovarian cancer cell lines over 72 hours. The data represents the mean of three replicates (+ SEM). The significance of differences in secreted TNF-α levels between treated and untreated (NT) cells was assessed using a two-tailed T-test;*** p<0.0001.

The earliest reports of taxane-induced TNF-α production involved the treatment of mouse macrophages with paclitaxel [37]. We have recently shown that MCF-7 (breast) and A2780 (ovarian) tumor cells exhibited increased production of TNF-α, 48 hours after treatment with 3 to 45 nM docetaxel [23]. In this study, it was determined that 2.5 nM docetaxel induced TNF-α release up to 96 hours after administration (Fig 1A and 1B). A dose response curve was generated at the 72 hour time point for each cell line (Fig 1C and 1D). We observed that both the concentration of drug and the exposure time are important factors affecting TNF-α release by docetaxel in both breast and ovarian cancer cell lines. It was also found that A2780 cells expressed considerably higher levels of TNF-α in response to docetaxel than MCF-7 cells, with the peak TNF-α concentrations greater than ten-fold above those observed in MCF-7 cells (Fig 1C and 1D).

#### A variety of classes of chemotherapy drugs induce TNF-α release from breast and ovarian tumor cell lines

**Fig 2:**
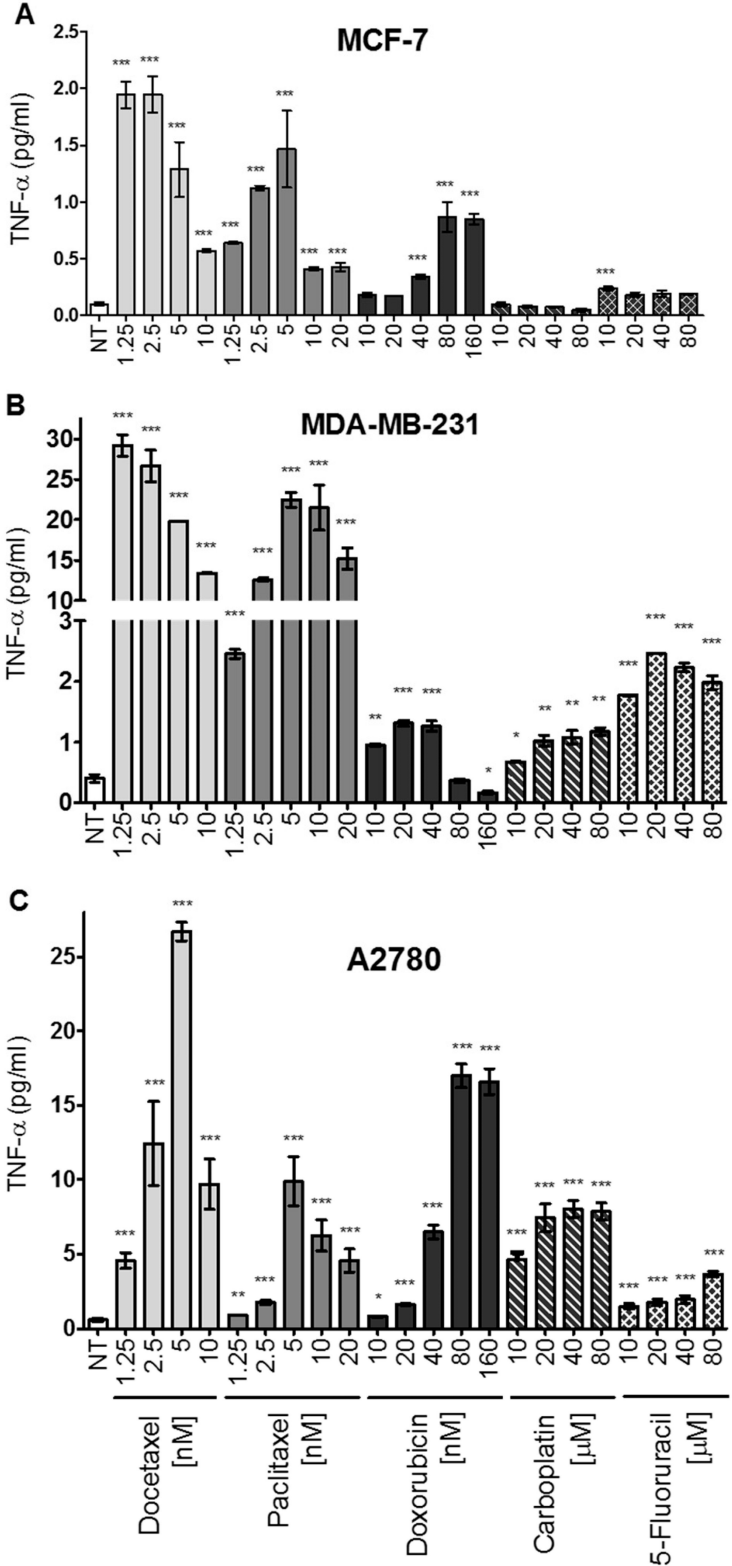
Increased TNF-α levels induced in select tumor cell lines by a variety of chemotherapeutic drugs. Soluble TNF-α levels in the media were measured by ELISA after 72 hr of treatment with either docetaxel, paclitaxel, doxorubicin, carboplatin, or 5-FU. Drug concentrations tested were based on IC_50_ values previously determined experimentally in MCF-7 cells. The data represents the mean of three replicates (+/-SEM). The significance of differences in TNF-α levels between treated and untreated cells was assessed using a two-tailed T-test; *** p<0.0001, **p<0.001, *p<0.05.

Members of several different classes of chemotherapy agents such as the taxanes (docetaxel and paclitaxel), the anthracycline doxorubicin, the platinating agent carboplatin, and the thymidine analog 5-Fluorouracil (5-FU) were used to treat various tumor cell lines for 72 hours at various concentrations. The levels of TNF-α released into the medium were then assessed using a TNF-α ELISA. Given that the concentration of docetaxel that optimally induced TNF-α release in MCF-7 and A2780 cells at 72 hours was approximately five-fold higher than the IC_50_, the concentrations used for testing the effects of other drugs were chosen relative to their published IC_50_ values in a previous study using MCF-7 cells [35]. MCF-7 cells responded to docetaxel, paclitaxel, doxorubicin, and 5-FU with significant increases in TNF-α levels. Carboplatin was without effect. However, MDA-MB-231 and A2780 tumor cells responded to all of the agents with statistically significant increases in secreted TNF-α levels. Both MDA-MB-231 and A2780 cells secreted more TNF-α than MCF-7 cells. Overall, docetaxel was the chemotherapy drug with the greatest capacity to induce TNF-α release in the cell lines tested (Fig 2A, 2B and 2C).

#### Increased media levels of TNF-α are due to increased transcription, not loss of membrane integrity

**Fig 3:**
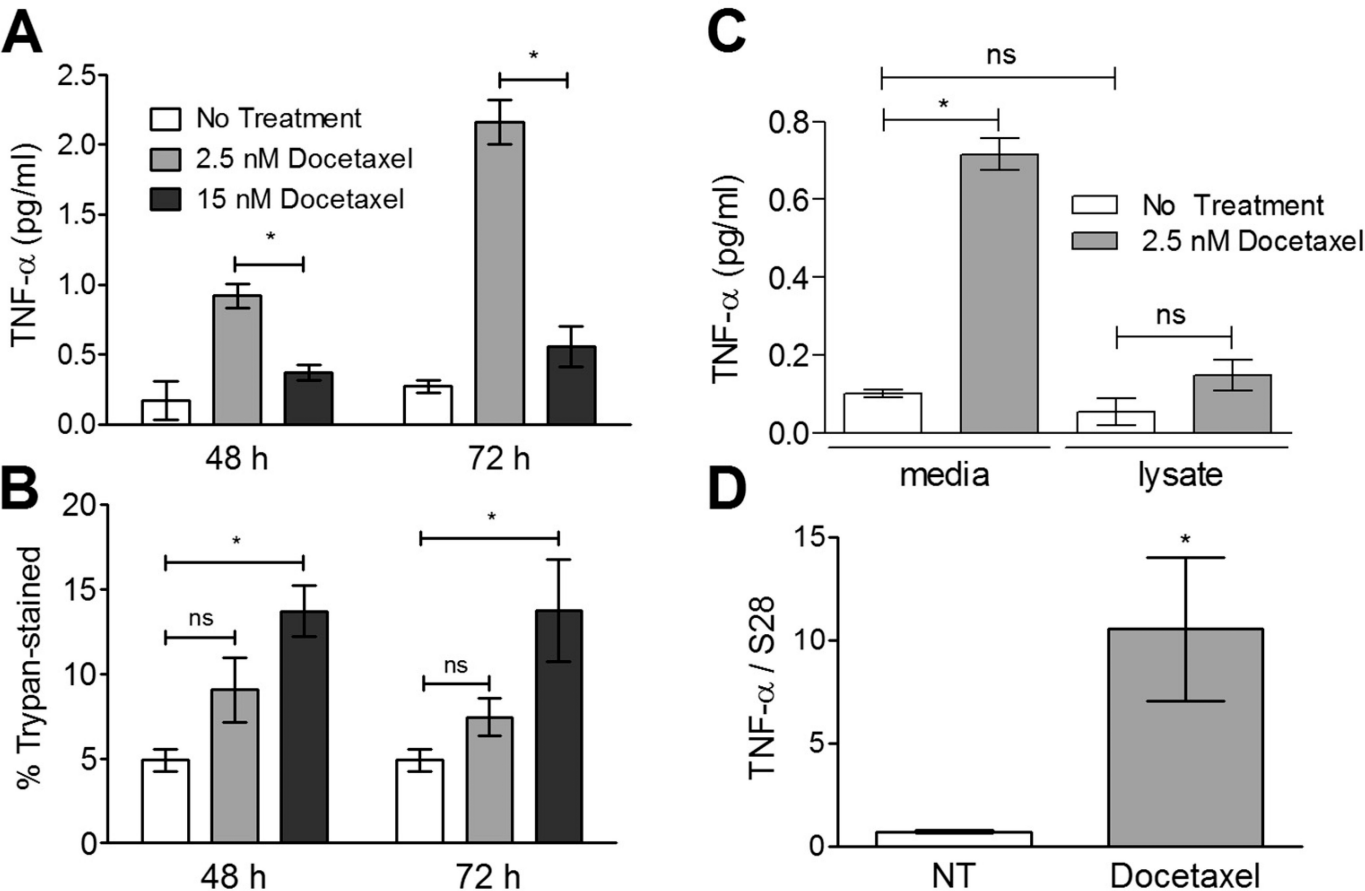
TNF-α release does not correlate with loss of membrane integrity. Cells were treated for 48 or 72 hours with one of two concentrations of docetaxel and the level of TNF-α in the media was measured (A) as well as the number of trypan-positive cells (B). The significance of differences in TNF-α levels or loss of membrane integrity were assessed using a 2-way ANOVA with a Tukey post-test. Cells were also lysed under hypotonic conditions and media levels of TNF-α from lysed and non-lysed were measured using an ELISA (C). TNF-α mRNA levels were also determined by quantitative PCR using cDNA generated from RNA extracts after 36 hours of docetaxel treatment. In these studies the significance of differences in TNF-α transcript levels between preparations was measured using an unpaired, two-tailed T-test; * p<0.05. All data represent the mean of three replicates (+/-SEM).

We assessed whether TNF-α levels had increased in the media due to the release of intracellular TNF-α following plasma membrane damage upon chemotherapy treatment. MCF-7 cells were treated for 48 or 72 hours with docetaxel at two different concentrations and the number of trypan blue-positive cells were quantified as a measure of lost plasma membrane integrity (Fig 3B). All docetaxel treatments caused an increase in the number of trypan blue-positive cells (Fig 3B). As expected the higher docetaxel concentration (15 nM) resulted in a higher number of trypan blue-positive cells than 2.5 nM docetaxel. In contrast, treatment with 15 nM docetaxel was not associated with the highest level of TNF-α in the media (Fig 3A), suggesting that TNF-α release is not principally associated with drug-induced cytolysis. In order to confirm this interpretation, cells were subjected to hypotonic conditions causing cell-lysis and assessed for the levels of TNF-α release (Fig 3C). Consistent with the previous interpretation, cell lysis alone did not result in significant increases in secreted TNF-α levels.

It has been shown that paclitaxel can induce increased production of TNF-α transcripts in macrophages [37]. We thus examined whether the drug-induced increase in media levels of TNF-α in MCF-7 cells involved the increased transcription of the human TNF-α gene. We observed that after 36 hours of exposure to 2.5 nM docetaxel, MCF-7 cells significantly increased their production of TNF-α transcripts by more than ten-fold (Fig 3D), while at earlier time points no significant increase was observed (data not shown).

### Comparing cellular responses to docetaxel and lipopolysaccharides in terms of TNF-α production

The idea that taxanes can activate an inflammatory pathway has been the focus of much study. This class of drug has often been shown to have 'LPS-mimetic' effects, as it has been reported that paclitaxel induces similar changes in gene expression to LPS [34]. LPS, also referred to as bacterial endotoxins, are the major structural components of the outer cell wall of Gram-negative bacteria. They are believed to be the cause of severe sepsis in patients with Gram-negative bacterial infections and it is suggested that most of the adverse effects of LPS stem from its ability to trigger the endogenous production of inflammatory cytokines [38]. Among the inflammatory cytokines, TNF-α is believed to be a primary mediator of sepsis, since direct infusion of animals with recombinant TNF-α produces most of the adverse events observed after LPS administration [39]. TLR4 is the primary receptor involved in LPS signaling. Activation of TLR4 by LPS has been shown to cause nuclear factor-κB (NF-κB)-mediated expression of TNF-α and CXCL8 [40], among other genes [41]. Another member of the TLR family (TLR2) has also been shown to mediate LPS signaling [42], although to a lesser extent. It has been suggested that the ability of taxanes to induce the production of inflammatory cytokines involves the *direct* activation of TLR4 [30,32,34,43,44]; however, clear evidence for this has yet to be demonstrated in tumor cells. Treatment of myeloid cells with LPS has been shown to result in the release of a variety of cytokines including TNF-α [30], CXCL8 [45], CXCL1 [46]. Further, each of these cytokines has been implicated in resistance to chemotherapy agents through either autocrine [23,24] or paracrine [25] signaling mechanisms. We thus assessed whether the tumor cell lines in this study were responsive to LPS as well as docetaxel, and whether these two agents stimulate the release of similar cytokines.

#### Cellular response to docetaxel is distinct from that of LPS in breast and ovarian cancer cells

**Fig 4:**
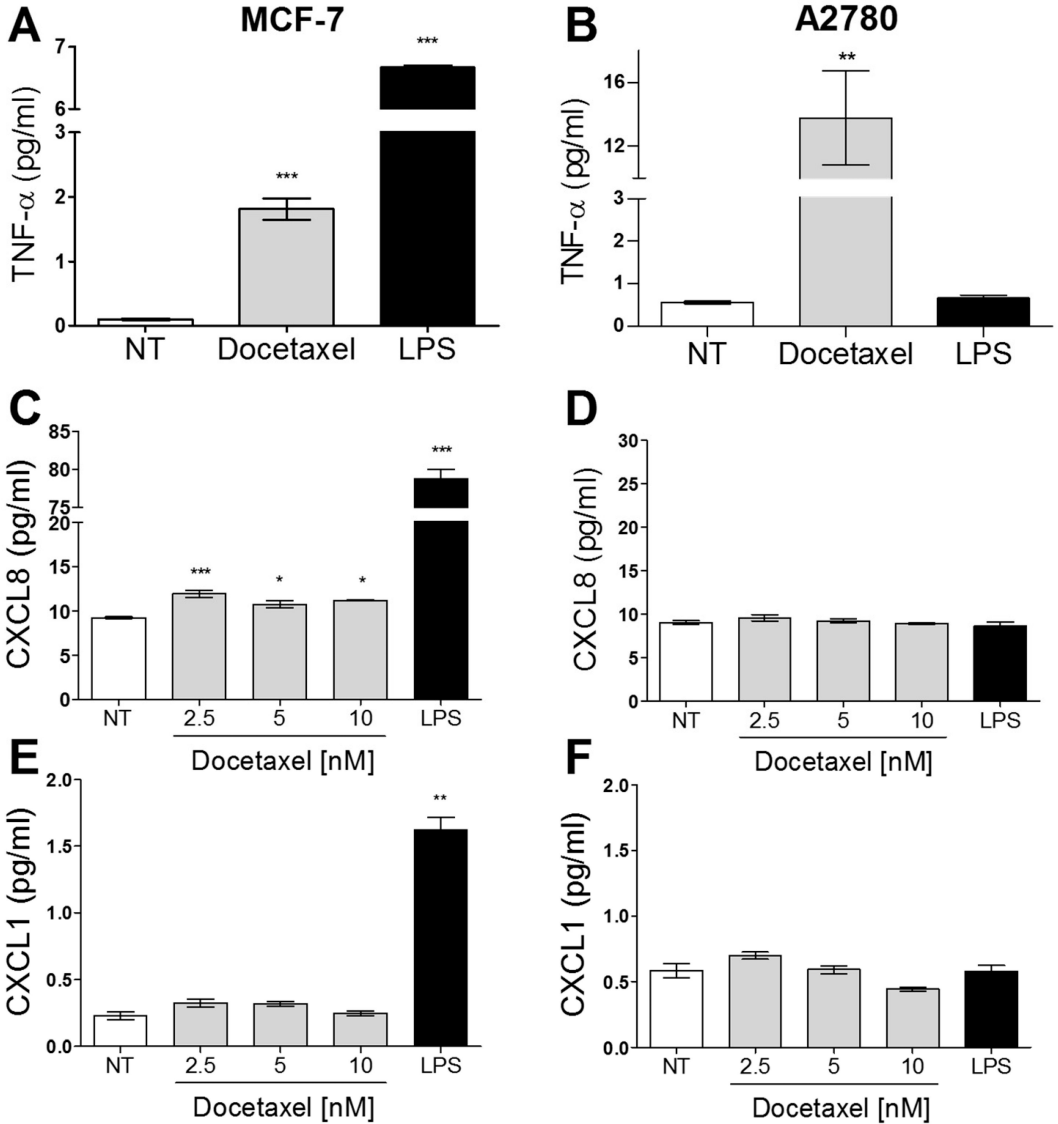
Media cytokine profiles for tumor cells after LPS or docetaxel exposure. The levels of TNF-α (A and B), CXCL8 (C and D), and CXCL1 (E and F) in cell media of either MCF-7 (A, C and E) or A2780 (B, D and F) cells were measured by ELISA after 72 hours of LPS or docetaxel treatment. The data represents the mean of three replicates (+/-SEM). All treatments were 10 μg/ml and 2.5 nM for LPS and docetaxel, respectively; The significance of differences in TNF-α levels between treated and untreated cells were determined using a two-tailed T-test; *** for p<0.0001, ** for p<0.01, and * for p<0.01.

**Fig 5:**
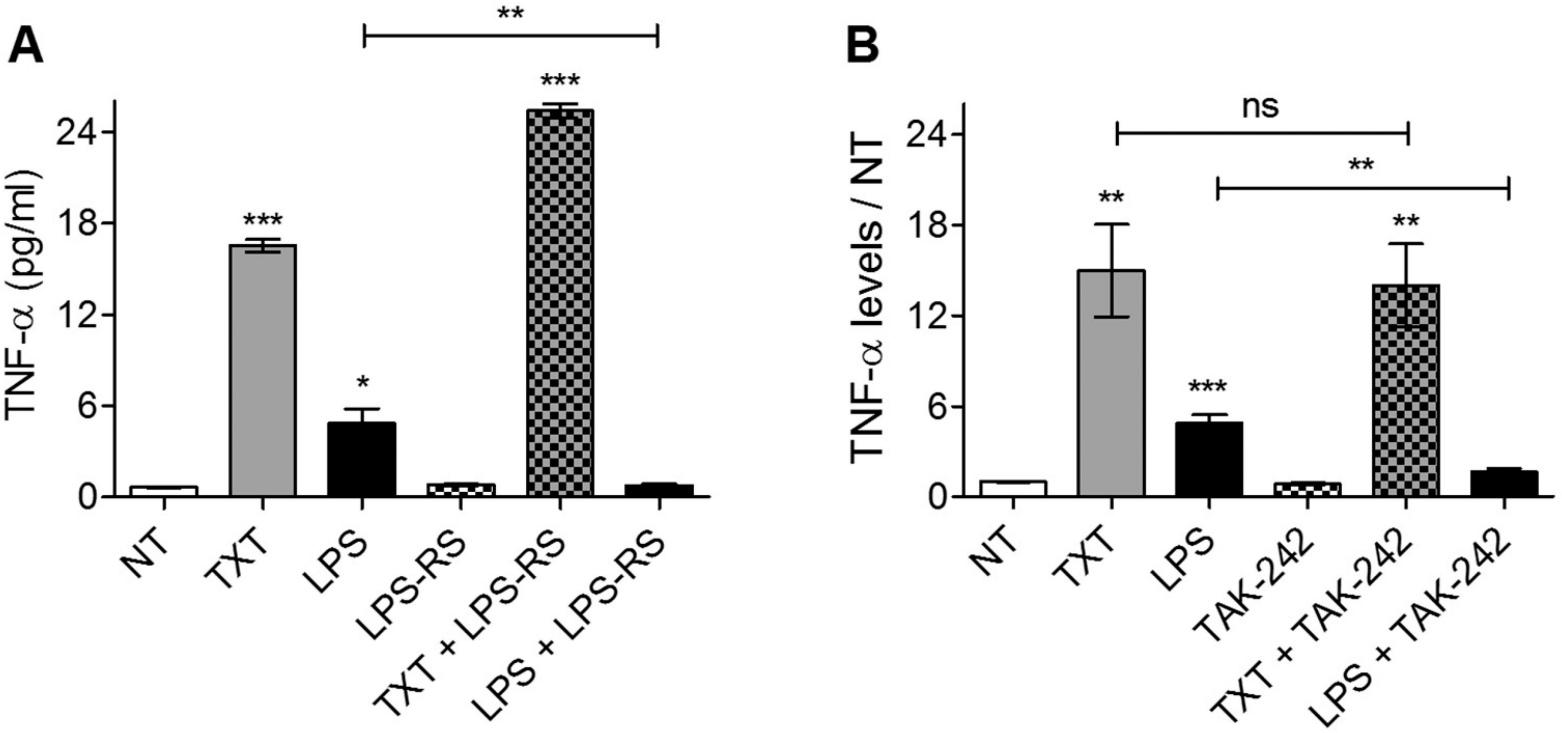
The effect of TLR4 inhibition on secreted TNF-α levels induced by docetaxel or LPS. MDA-MB-231 cells were treated for 72 hours with either 2.5 nM docetaxel (TXT) or 0.1 μg/ml LPS, after pretreatment with either 100 μg/ml LPS-RS (A) or 0.1 μg/ml TAK-242 (B). The data represent the mean of three replicates (+/-SEM). The significance of differences between treated and untreated cells were assessed using a two-tailed T-test; ***p<0.001, **p<0.01, *p<0.02.

MCF-7 cells responded to LPS with the release of TNF-α, CXCL8, and CXCL1. In contrast, docetaxel only induced detectable increases in TNF-α and CXCL8 expression. A2780 cells, in contrast, responded to docetaxel with the release of TNF-α only, and when challenged with LPS, no significant changes in any of the above cytokines were observed (Fig 4). Unlike the canonical (hexa-acylated) form of LPS produced in E. coli, under-acylated forms such as tetra- and penta-acylated LPS (lipid IVA) are fundamentally distinct in their interactions with TLR4. Rather they are antagonists of TLR4 activation [47]. Penta-acylated LPS is synthesized by some bacteria, including Rhodobacter Sphaeroides [48] and elicits an inhibitory effect through the binding of the adaptor protein myeloid differentiation factor 2 (MD-2), forming a complex that suppresses TLR4 activation [47,49,50]. Recently, another group has shown that TLR4 activity can also be selectively inhibited by a small molecule called TAK-242 [51]. Unlike LPS-RS, TAK-242 associates with the intracellular TIR (toll-interleukin-1 receptor) domain of TLR4 and prevents its association with intracellular adaptor proteins. It was therefore of interest to determine whether inhibition of TLR4 signaling with either penta-acylated LPS from R. Sphaeroides (LPS-RS) or TAK-242 would inhibit docetaxel-induced TNF-α release in tumor cells. Using MDA-MB-231 cells (prominent TLR4 expressers) it was found that treatment with an LPS-RS concentration of 100-fold higher than that of LPS caused complete inhibition of LPS-induced TNF-αrelease, consistent with experiments in human monocytes [48]. However, LPS-RS did not suppress docetaxel's ability to induce TNF-α release, but rather augmented it (Fig 5A). In a similar experiment the TLR4 antagonist TAK-242 was administered in combination with either docetaxel or LPS (Fig 5B). As with LPS-RS, TAK-242 completely abrogated LPS-induced TNF-α release from MDA-MB-231 cells; however, it did not significantly affect docetaxel-induced TNF-α release. Together these findings demonstrate that interference with the LPS-binding domain (Fig 5A) or blocking the association between the intracellular TIR domain of TLR4 and its adaptor molecules is not able to prevent docetaxel-induced TNF-α release, while in both cases abrogating that induced by LPS. Moreover, since there have been reports that LPS can activate cellular processes dependent upon other toll-like receptors [for example, TLR2 [52]] and since TLR4 can form heterodimers with TLR2 [53], our observations with the TLR4-specific inhibitor TAK-242 suggest that LPS-induced TNF-α production is through the ability of LPS to activate TLR4 and not other toll-like receptors.

#### Inhibition of docetaxel-induced TNF-α release by Marimastat

In macrophages it is thought that TNF-α release, particularly in response to LPS, occurs by constitutive exocytosis, involving a receptor-mediated increase in gene transcription [54,55]. Increased gene transcription results in increased production of a membrane-bound precursor of TNF-α (mTNF-α), and TNF-α release involves cleavage of mTNF-αto produce the soluble form (sTNF-α) [56]. Hydroxamate-based agents cause inhibition of a broad-spectrum of matrix metalloproteinases (MMPs), and have also been shown to inhibit TNF-α release, suggesting that one or more MMPs are involved in this process, including TNF-α-converting enzyme (TACE, also known as ADAM-17) [56]. Although the pathways governing cytokine trafficking and release have been well-studied in macrophages and in other murine cell lines, there is a lack of understanding about the pathways responsible for cytokine trafficking in epithelial cells [54] or in tumor cells of epithelial origin. It was therefore of interest to determine whether similar mechanisms are employed during the processing of drug-induced TNF-α release in epithelial-derived tumor cell lines. We thus examined the effect of the hydroxamate-based MMP inhibitor Marimastat on LPS- and docetaxel-induced TNF-α release from various epithelial-derived cell lines.

**Fig 6:**
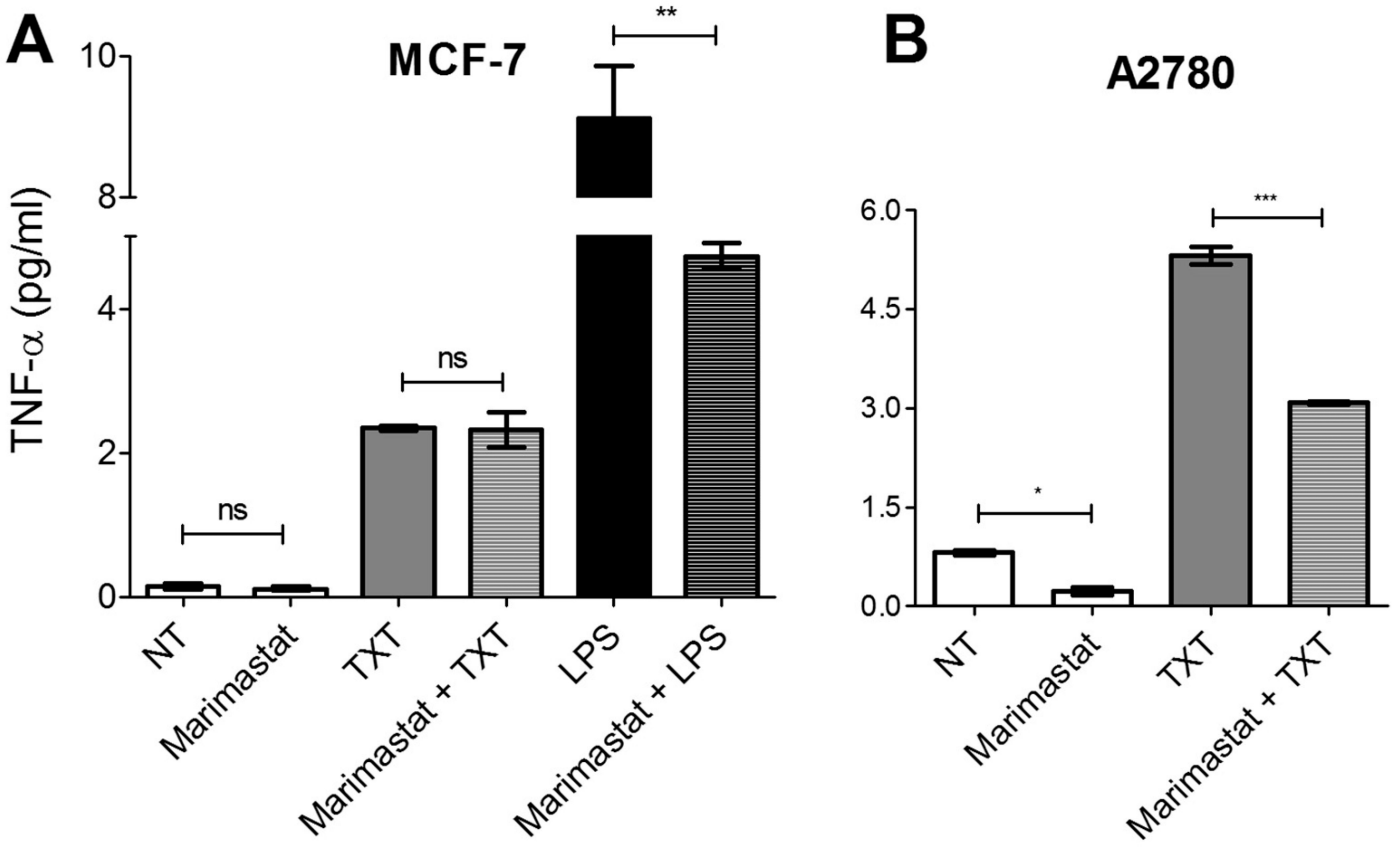
The effect of MMP inhibition by Marimastat on cellular TNF-α levels. MCF-7 and A2780 cells were treated with 2.5 nM docetaxel (TXT) or 10 μg/ml LPS for 72 hours in the presence or absence of 200 nM Marimastat, a broad-spectrum MMP inhibitor. The data represent the mean of three replicates (+/-SEM). The significance of differences in TNF-α levels between treatments was assessed using a two-tailed T-test; *** p<0.0001, ** p<0.001, *p<0.01.

Marimastat, which reportedly inhibits MMP-1, 2, 3, 7, 8, 9, and 14, as well as ADAM-17 with respective IC_50_ values of 5, 6, 200, 20, 2, 3, 1.8, 3.8 nM [57], did not have a significant effect on docetaxel-induced TNF-α release from MCF-7 cells (Fig 6A). In contrast, LPS-induced TNF-α release was significantly diminished (by ~50%) in the presence of Marimastat. Our findings suggest that in MCF-7 cells LPS-induced TNF-α release is mediated, at least in part, by shedding of TNF-α from the plasma membrane by MMPs. It was, however, unclear whether MMPs play a role in docetaxel-induced TNF-α release from MCF-7. Supporting the role of MMPs in TNF-α release, we observed that Marimastat inhibits basal and docetaxel-induced TNF-αrelease from A2780 cells (Fig 6B). The differential sensitivity to Marimastat suggests that the mechanisms for docetaxel-induced TNF-α release in MCF-7 and A2780 cells are distinct.

### Changes in TNF-α production upon selection for docetaxel resistance

#### P-glycoprotein (Abcb1) mediates the docetaxel-resistant phenotype

A series of docetaxel-resistant breast tumor cell lines (MCF-7_TXT7_ to MCF-7_TXT12_ cells) were previously created by selection of wild-type MCF-7 cells for survival in increasing concentrations of docetaxel [35]. These cells were shown to exhibit progressively increased transcription of the gene encoding P-glycoprotein (P-gp, also known as Abcb1) relative to cells “selected” in the absence of drug (co-cultured MCF-7_CC_ cells) [58]. We thus assessed whether increased ABCB1 gene transcription was associated with corresponding increases in P-gp protein levels.

**Fig 7:**
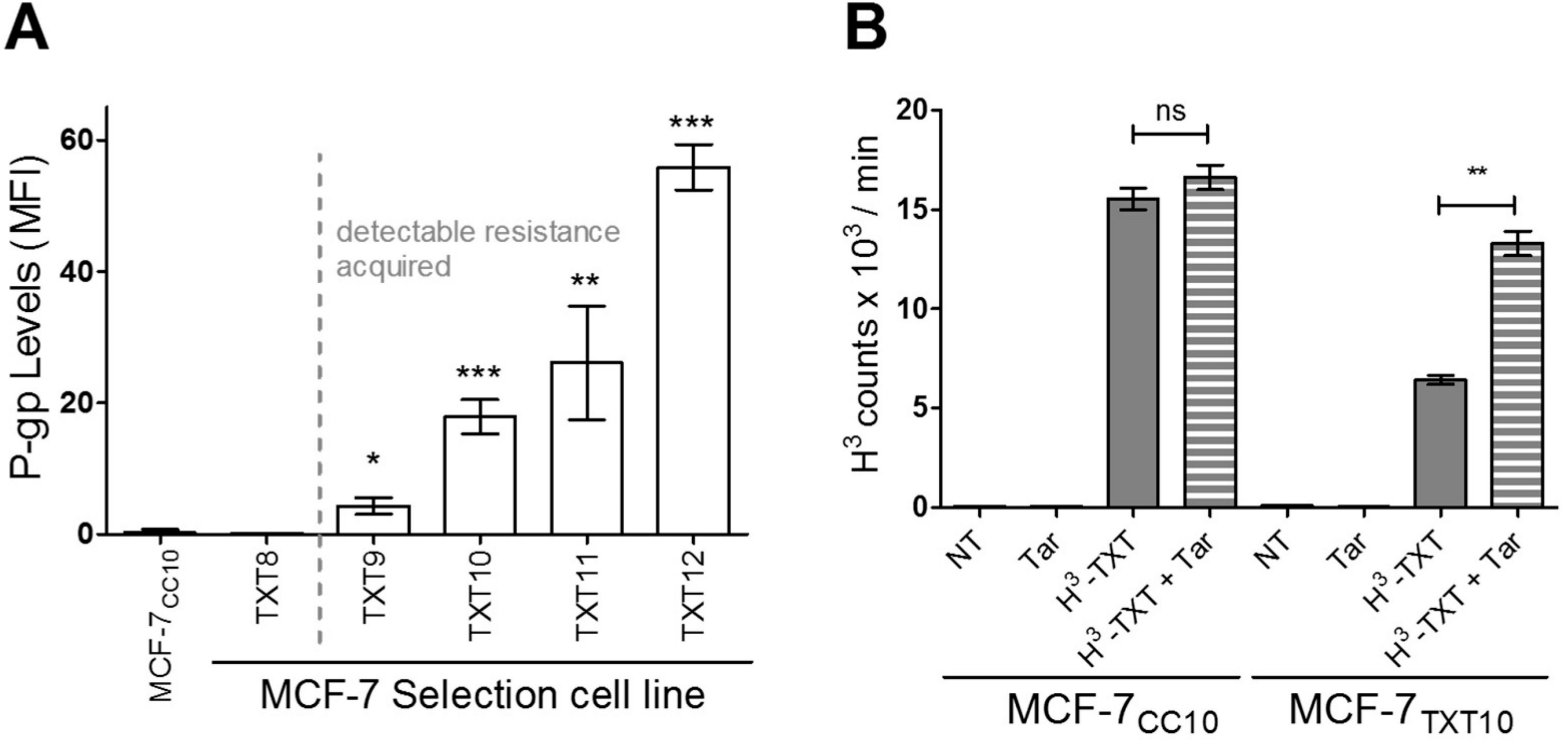
Increased P-gp levels are associated with diminished drug uptake in drug-resistant MCF-7 cells. (A) Flow cytometry was used to assess P-gp levels [as expressed by mean fluorescence intensity (MFI) values] for MCF-7 cells at selection doses 8, 9, 10, 11, and 12. (B) Docetaxel-resistant MCF-7_TXT10_ cells exhibited decreased intracellular drug accumulation, which was restored with the addition of Tariquidar (Tar), an allosteric inhibitor of P-gp. Tritiated docetaxel (H3-TXT) was administered at a concentration of 2.5 nM, either with or without 100 nM Tariquidar for 12 hours and docetaxel uptake was determined by measuring the radioactivity of cells. All data represents the mean of 3 replicates (+/-SEM). Two-tailed T-tests were employed to assess the significance of differences in docetaxel uptake between the various treatments in the control (MCF-7_CC10_) and docetaxel-resistant (MCF-7_TXT10_) cell lines; *** for p<0.0001, **p<0.001, and * for p<0.01.

As shown in Fig 7A, MCF-7_TXT_ cells exhibited progressively increased P-gp protein expression (relative to the MCF-7_CC10_ cell line), which correlated with their observed level of docetaxel resistance. Detectable increases in P-gp protein expression (relative to MCF-7_CC_ cells) first occurred at selection dose 9 (MCF-7_TXT9_ cells), which was also the selection dose at which docetaxel resistance was first achieved. Consistent with P-gp’s ability to transport docetaxel [59] and other chemotherapy drugs out of tumor cells [60], we observed that docetaxel-resistant MCF-7 cells showed decreased uptake of tritium-labeled docetaxel (H^3^-TXT), relative to the drug-sensitive MCF-7_CC10_ cell line (Fig 7B). In order to assess whether P-gp was responsible for the decreased docetaxel uptake, a specific allosteric inhibitor for P-gp, Tariquidar, was given in combination with docetaxel (Fig 7B). It was found in MCF-7_TXT10_ cells that drug uptake was restored significantly upon addition of Tariquidar, thus confirming the role of P-gp in the export of docetaxel from drug-selected cells.

**Fig 8:**
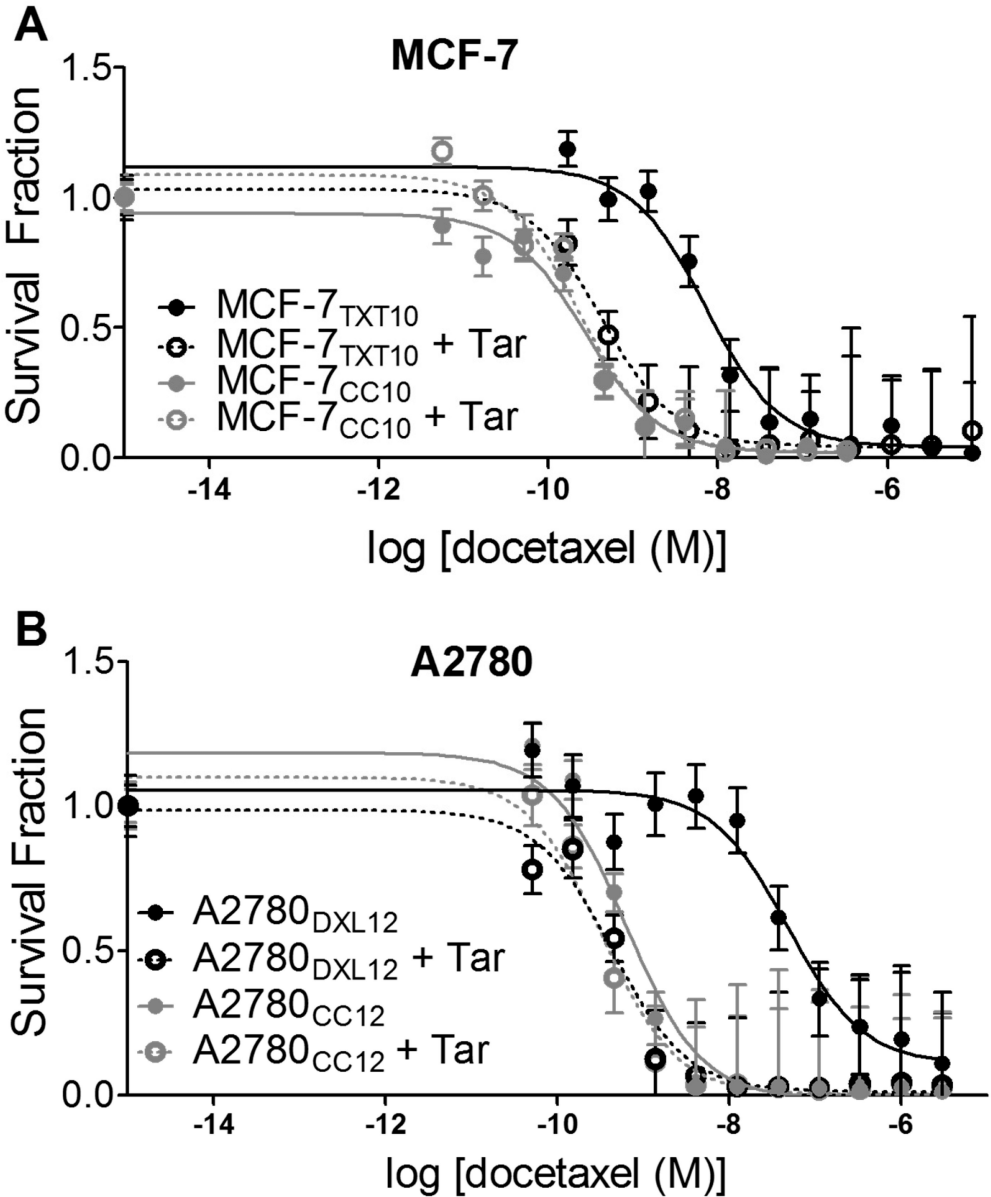
Sensitivity to docetaxel is restored upon inhibition of P-gp activity with Tariquidar (Tar). (A) Clonogenic assays yielded IC_50_ values for docetaxel in MCF-7_CC10_ cells of 0.28 nM and 0.24 nM, with and without 100 nM Tariquidar, respectively. IC_50_ values for MCF-7_TXT10_ cells were significantly different with (0.42 nM) and without (7.14 nM) Tariquidar, respectively (p<0.0001). (B) An identical trend was observed for the A2780_CC12_ (IC_50_ = 0.630 nM) and A2780_DXL12_ (IC_50_ = 47.5 nM) cell lines, where in the presence of Tariquidar IC_50_ values were 0.331 nM and 0.435 nM, respectively. Each data point represents the mean number of colonies (+/-SEM) in twelve independent microscopic fields. Each experiment was replicated three times with consistent trends. Non-linear regression analysis was used to compare the significance of difference in IC_50_ values between Tariquidar-treated and untreated conditions.

Given that increased P-gp levels were shown to affect docetaxel uptake in docetaxel-resistant cells, we assessed whether restoration of drug-uptake would also restore docetaxel sensitivity. As shown in Fig 8A and 8B, pre-incubation of docetaxel-resistant MCF-7_TXT10_ and A2780_DXL12_ cells with the P-gp inhibitor Tariquidar increased sensitivity to docetaxel, while having no significant effect on drug sensitivity in the co-cultured control cell lines at their respective selection doses. The ability of Tariquidar to restore drug sensitivity in drug-resistant cells to a level that is close to their respective co-cultured control cells suggests that the docetaxel-resistant phenotype is primarily achieved by P-gp–mediated drug efflux from the cells.

#### Basal production of multiple cytokines increases in tumor cell lines selected for resistance to docetaxel

Previous reports by our laboratory have shown that MCF-7 cells selected for resistance to docetaxel exhibited increased basal levels of TNF-α. At very high selection doses, TNF-α production eventually returned to the level of co-cultured control cells [23]. In the same study, it was reported that acquisition of docetaxel resistance in MCF-7 cells was associated with decreased expression of TNF-αreceptor 1 protein (TNFR1) relative to drug-sensitive control cells, thereby diminishing TNF-α’s ability to induce apoptosis. The increased TNF-α production was thought to contribute to the resistant phenotype by activating TNFR2-dependent survival pathways, although TNFR2 blockade accounted for only a small restoration of drug sensitivity relative to that achieved by inhibiting NF-κB [23]. Activation and subsequent nuclear localization of NF-κB can occur in response to a variety of extracellular stimuli, including endogenously produced inflammatory cytokines such as TNF-α, CXCL8 [61], and CXCL1 [62]. Thus, we assessed whether increases in TNF-α, CXCL8, and CXCL1 would also be observed during selection of tumor cells for survival in increasing concentrations of docetaxel.

**Fig 9:**
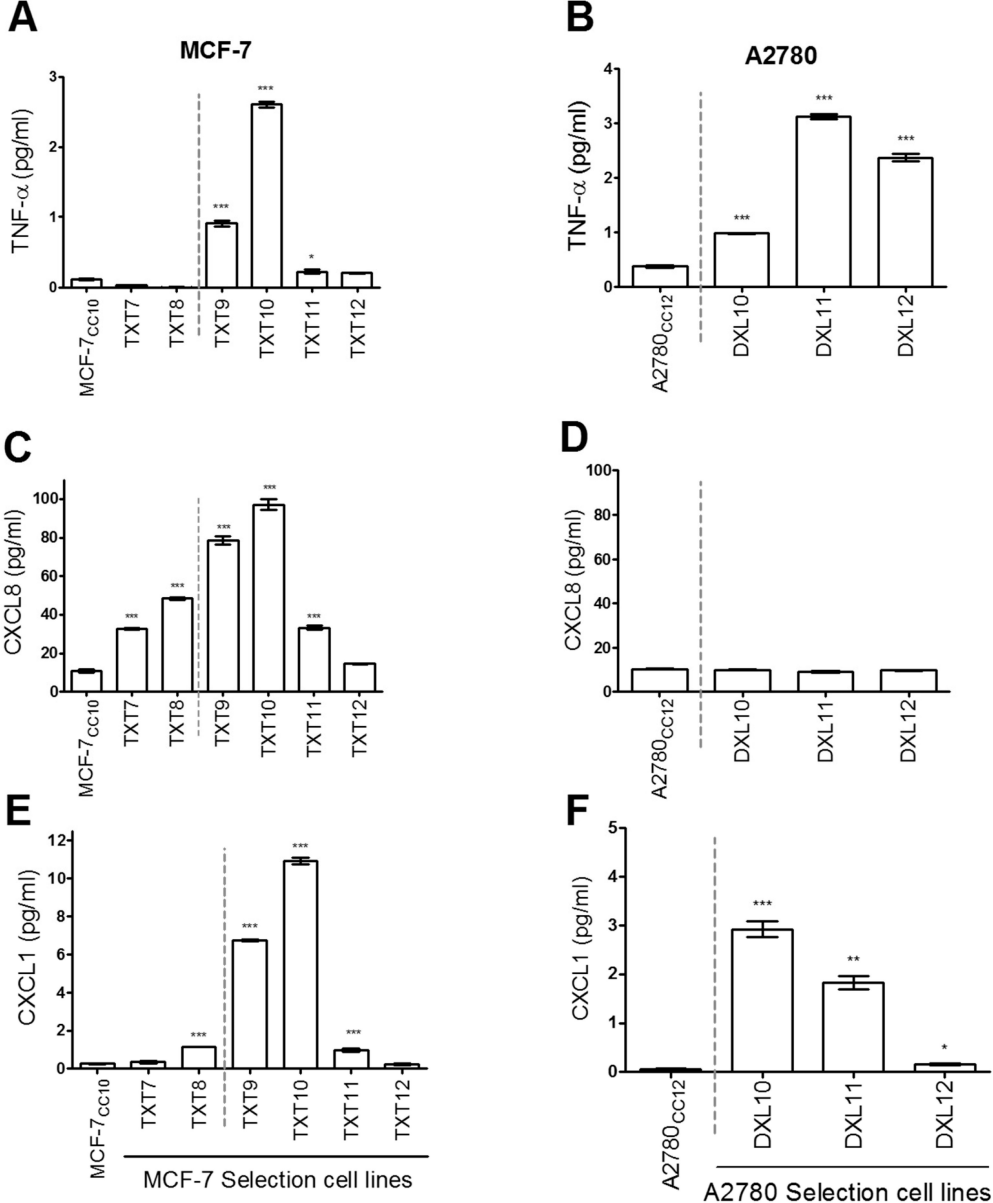
Basal cytokine production changes during selection for resistance to docetaxel. Levels of TNF-α (A and B), CXCL8 (C and D), and CXCL1 (E and F) in media as measured by ELISA, from MCF-7 (A, C, and E) and A2780 cells (B, D, and F) after 72 hours of cell culture. Cell lines to the right of the vertical broken grey line exhibit acquired resistance to docetaxel, as confirmed in clonogenic assays. Each value represents the mean of three replicates (+/-SEM). Two-tailed T-tests were used to assess the significance of differences in cytokine levels between docetaxel-selected cell lines and their respective co-cultured control cell lines (MCF-7_CC10_ or A2780_CC12_); ***p<0.0001, **p<0.001, *p<0.01.

As shown in Fig 9, basal TNF-α levels increased upon selection for docetaxel resistance, when the selection dose reached level 9 and 10 (MCF-7_TXT9_ and MCF-7_TXT10_ cells), beyond which selection at higher doses resulted in TNF-α levels subsiding back to that of non-selected cells. Similarly, it was also found that basal levels of CXCL8 and CXCL1 increased with maximum production in MCF-7_TXT10_ cells, beyond which their levels also declined toward those of non-selected cells (Fig 9). Interestingly, A2780_DXL_ cells above selection dose 10 showed a similar elevation in basal TNF-αand CXCL1 levels (Fig 9B and 9F); however, no changes in CXCL8 expression were observed (Fig 9D).

#### Elevated basal production of TNF-α in docetaxel-resistant cells is inhibited upon treatment with a broad-spectrum MMP inhibitor

**Fig 10:**
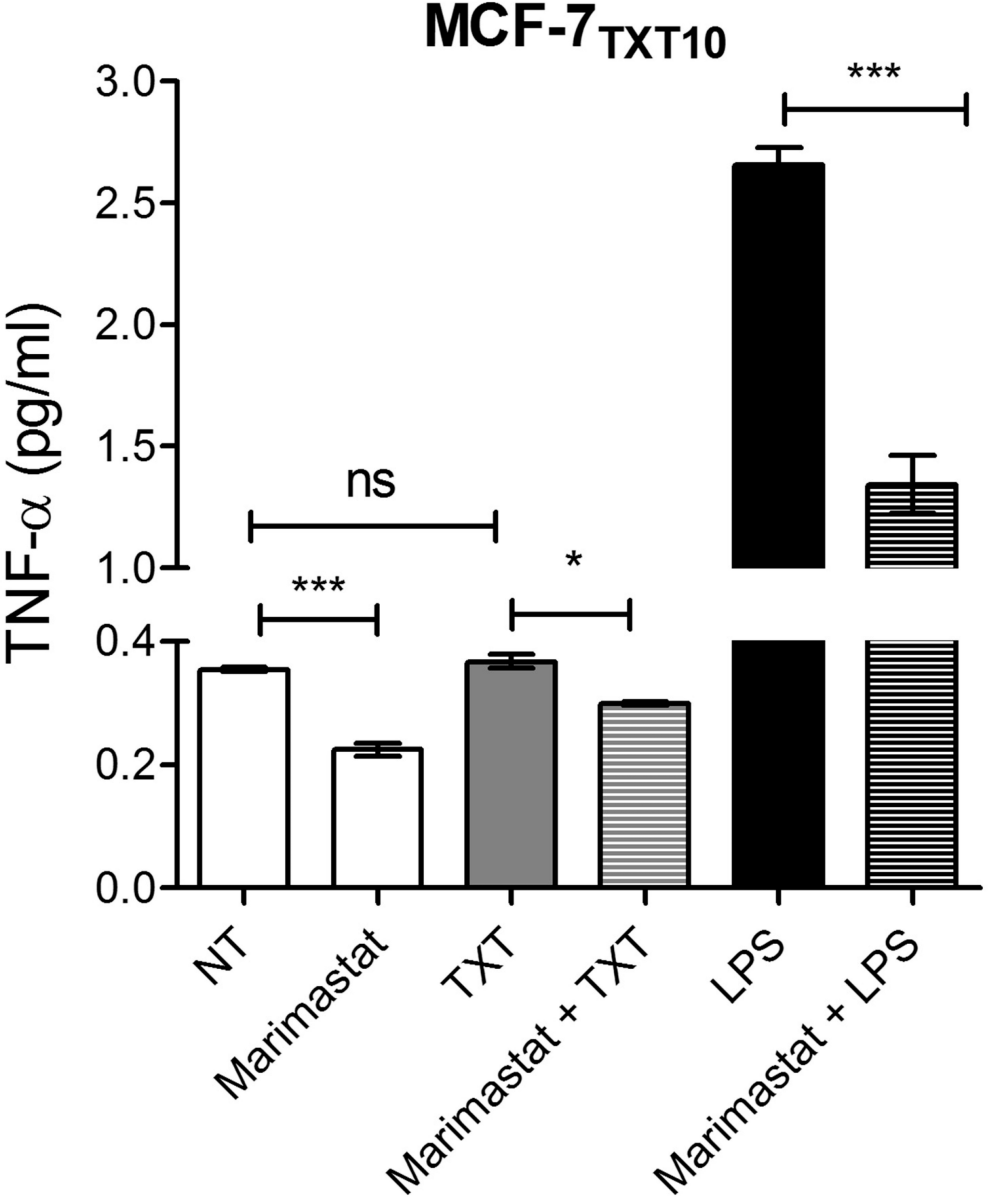
Effect of Marimastat on TNF-α levels in the medium of MCF-7_TXT10_ cells. Cells were treated for 72 hours with media only (NT), 200 nM Marimastat, 2.5 nM docetaxel (TXT), 10 μg/ml LPS, or a combination thereof. The data represents the mean of three replicates (+/-SEM) and the significance of differences in TNF-α levels between treatments was determined using a T-test; *p<0.01, **p<0.001, ***p<0.0001.

As mentioned, TNF-α is first synthesized as an integral membrane protein that is typically released from cells through the action of the metalloproteinase ADAM-17 [56]. We thus examined the effect of the MMP inhibitor Marimastat on the ability of docetaxel-resistant MCF-7 cells to produce elevated basal levels of TNF-α. As shown in Fig 10, Marimastat was able to inhibit basal TNF-α production in MCF-7_TXT10_ cells, suggesting that this process depends, at least in part, on MMP activity and shedding of membrane-bound TNF-α. Unlike the previous observations in the drug-naive MCF-7 cell line, the production of TNF-α in MCF-7_TXT10_ cells was not augmented by treatment with 2.5 nM docetaxel for 72 hours. In contrast to docetaxel, LPS was able to strongly augment TNF-α production, and this production was also inhibited by Marimastat. TNF-α release was not fully abolished by Marimastat under any conditions.

#### Differences in pathways leading to TNF-α release in tumor cells

**Fig 11:**
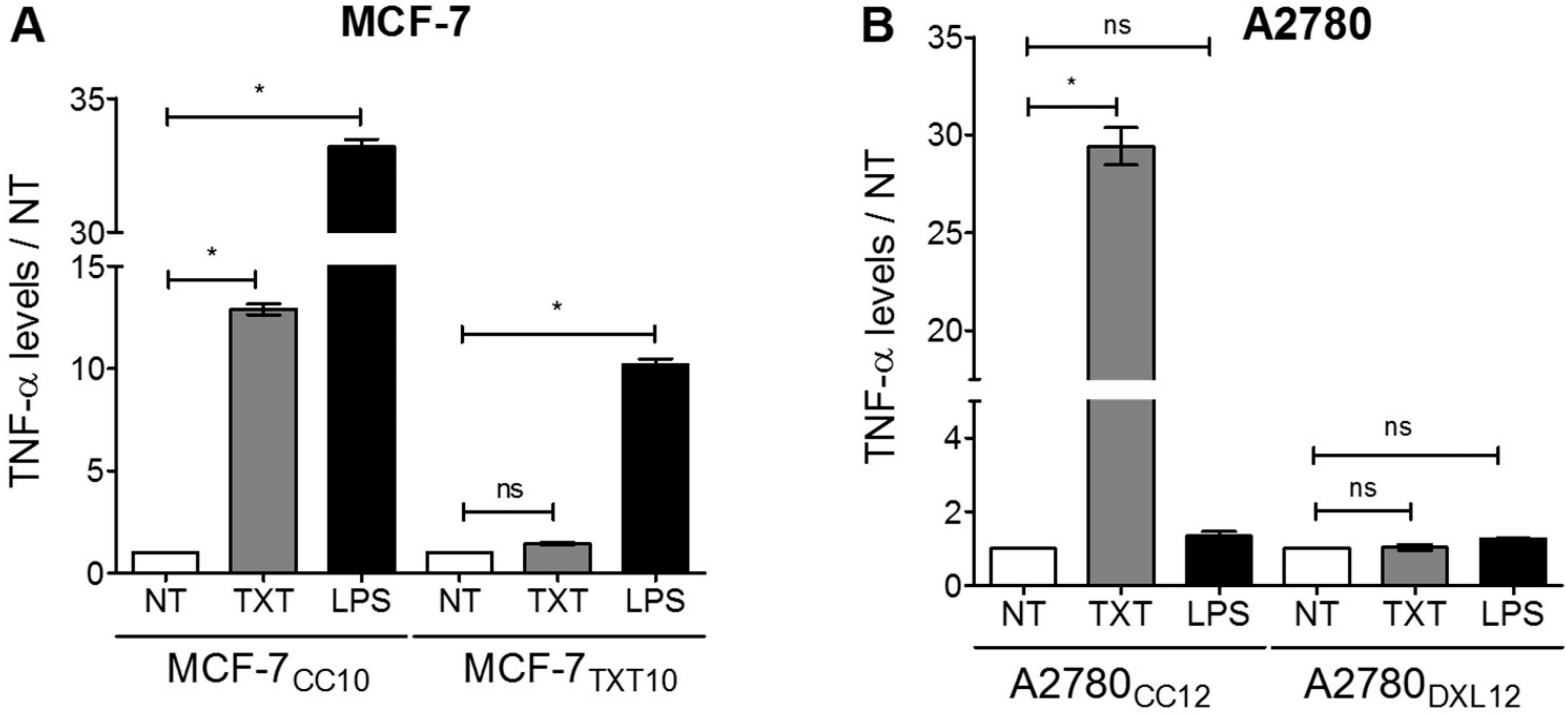
**Comparing the effects of docetaxel and LPS treatment on TNF-α release from drug-naive and drug-resistant cell lines for MCF-7 (A) and A2780 (B) cells.** All docetaxel (TXT) concentrations used were 2.5 nM and LPS concentrations were 5 μg/ml. Comparisons between treated and untreated TNF-α levels were assessed for significance using a one-way ANOVA with a Tukey post-test; * for p<0.05; all data points represent the mean of three trials.

**Fig 12:**
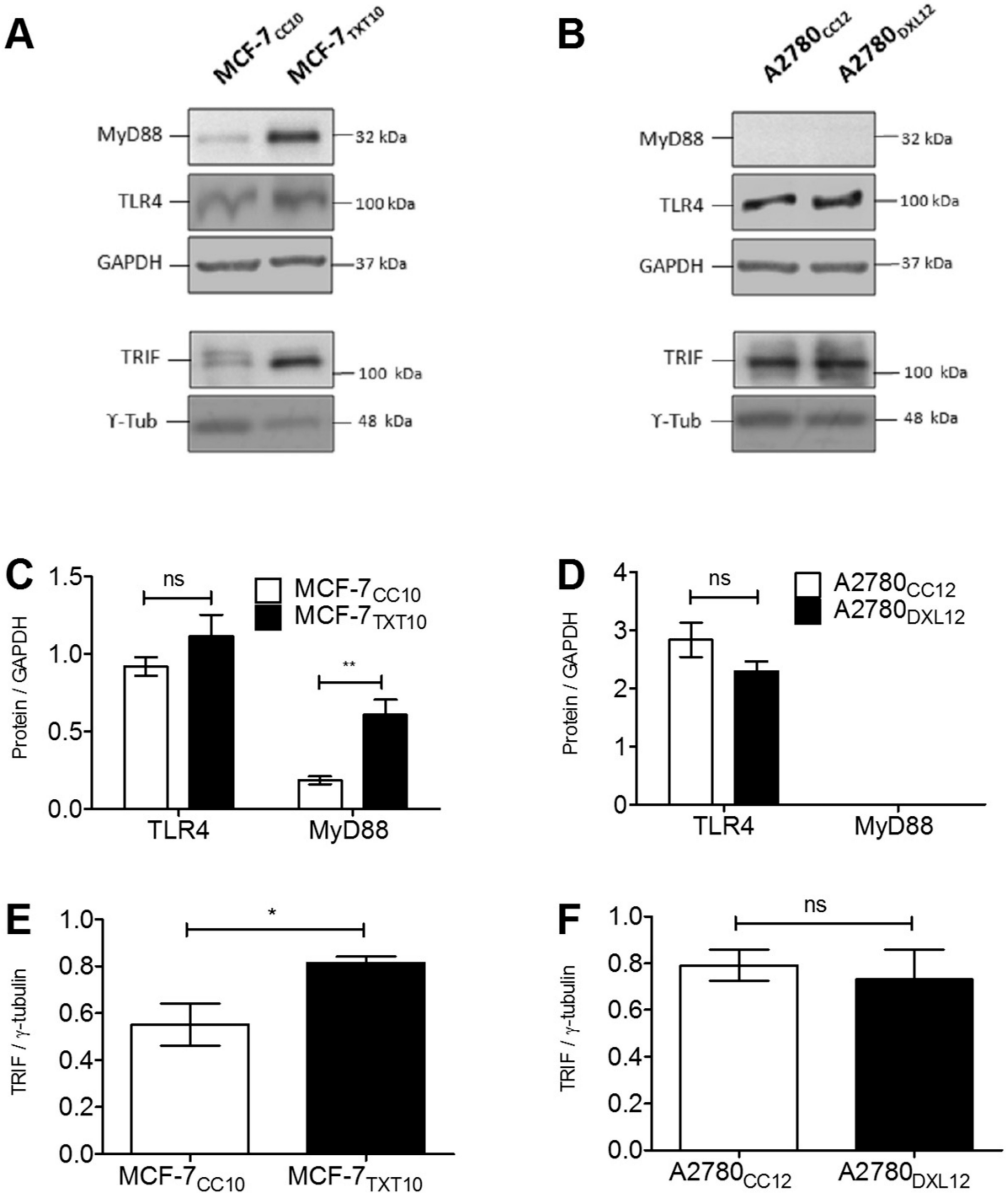
Levels of TLR4 and adaptor proteins during acquisition of resistance to docetaxel. Immunoblots were performed from extracts of MCF-7 and A2780 drug-naive and drug-resistant cell lines (A and B) in order to confirm the presence or absence of TLR4 and adaptor proteins MyD88 and TRIF. Changes in protein levels of drug-naive and drug-resistant cell lines were assessed by densitometry (C, D, E, and F). Statistical analysis consisted of two-tailed T-tests; *p<0.05.

Docetaxel-resistant MCF-7_TXT10_ and A2780_DXL12_ cells failed to increase their output of TNF-α in response to a 72-hour docetaxel exposure, in contrast to their respective co-cultured control cell lines. Remarkably, TNF-α release could be stimulated by LPS in both docetaxel-sensitive (MCF-7_CC10_) and docetaxel-resistant (MCF-7_TXT10_) cells (Fig 11A). In contrast, docetaxel-resistant A2780_DXL12_ cells remained unresponsive to LPS, along with respective co-cultured control cells (Fig 11B), as previously described (Fig 4). Immunoblot experiments revealed that both MCF-7 and A2780 cell lines expressed the cell-surface receptor TLR4 (Fig 12A, 12B, 12C and 12D). MyD88 (TLR4 adaptor protein) levels were detectable in MCF-7 cells and were elevated greater than three-fold upon acquisition of docetaxel resistance (MCF-7_TXT10_ cells) (Fig 12A and 12C). By contrast, MyD88 was undetectable in both A2780 and their docetaxel-resistant counterparts (A2780_DXL12_) (Fig 12B and 12D). Another TLR4 adaptor protein, TIR-domain-containing adapter-inducing interferon-β (TRIF) was detected in all four tumor cell lines and its expression increased in MCF-7_TXT10_ relative to the unselected MCF-7_CC10_ cells (Fig 12 A, 12B, 12E and 12F).

#### Restoring cellular drug accumulation potentiates drug-induced TNF-α release in docetaxel-resistant cells

**Fig 13:**
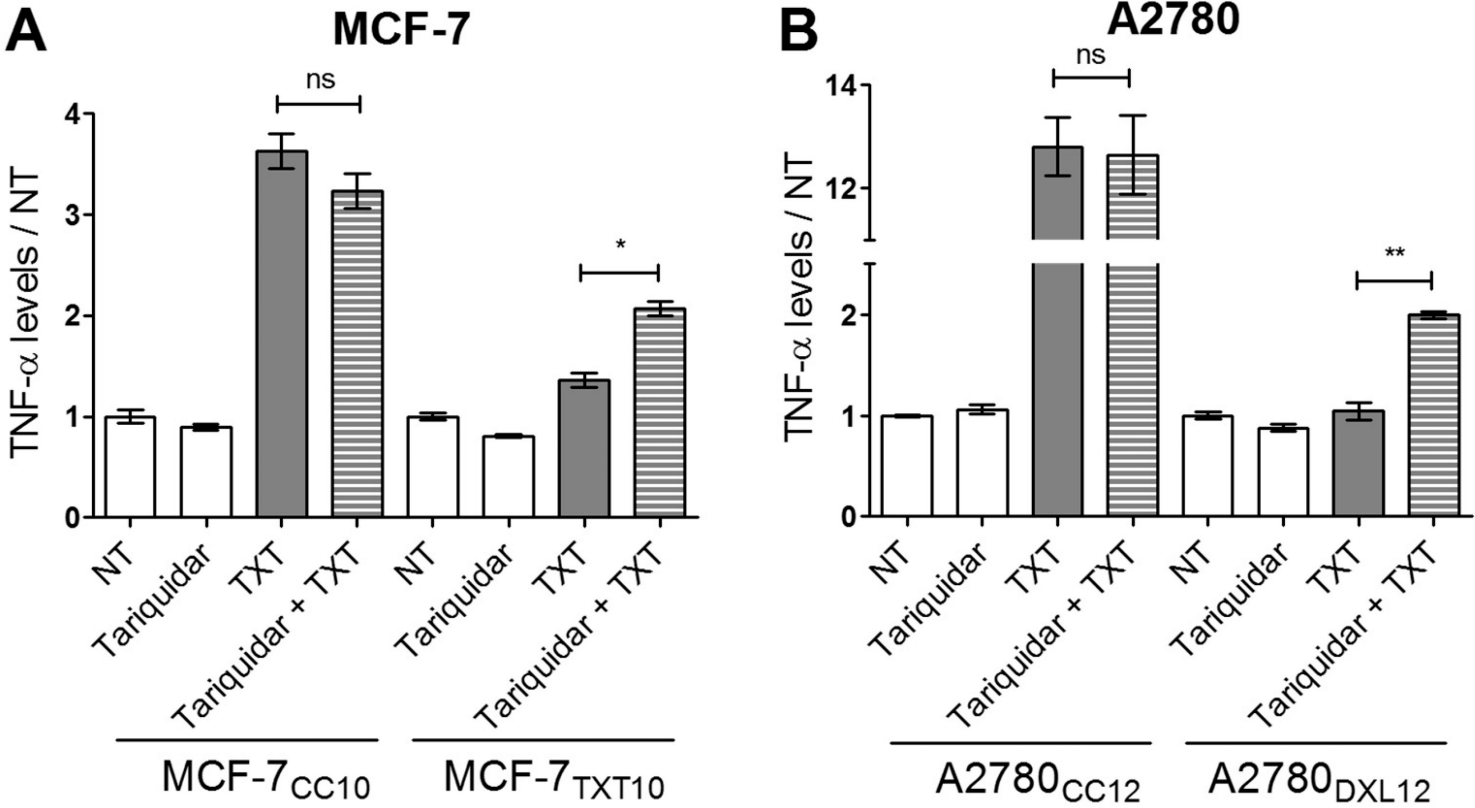
Effects of Tariquidar on docetaxel-induced TNF-α release. All treatments lasted 72 hours at which point levels of TNF-α in the media were measured by ELISA for MCF-7 (A) and A2780 (B) cells. Cells were either untreated or treated with 100 nM Tariquidar and/or 2.5 nM docetaxel (TXT). The data are representative of three replicates (+/-SEM). A two-tailed T-test was used to assess the significance of differences in cellular TNF-α production between treatments;** for p<0.001, * for p<0.01.

Given the presence of P-gp and its contribution to reduced cellular docetaxel accumulation in docetaxel-resistant, MCF-7_TXT10_ and A2780_DXL12_, cells (Fig 7), we examined the effect of the P-gp inhibitor Tariquidar on docetaxel-induced TNF-α production in these cell lines. As shown in Fig 13, pre-incubation with Tariquidar had no significant effect on drug-induced TNF-α release in co-cultured control MCF-7 and A2780 cells. In contrast, Tariquidar potentiated docetaxel-induced TNF-α release from MCF-7_TXT10_ and A2780_DXL12_ cells, suggesting that docetaxel accumulation within tumor cells (drug entry) is required for drug-induced TNF-α release.

### Effects of LPS pretreatment on docetaxel cytotoxicity

#### LPS augments docetaxel cytotoxicity in breast tumor cells

**Fig 14:**
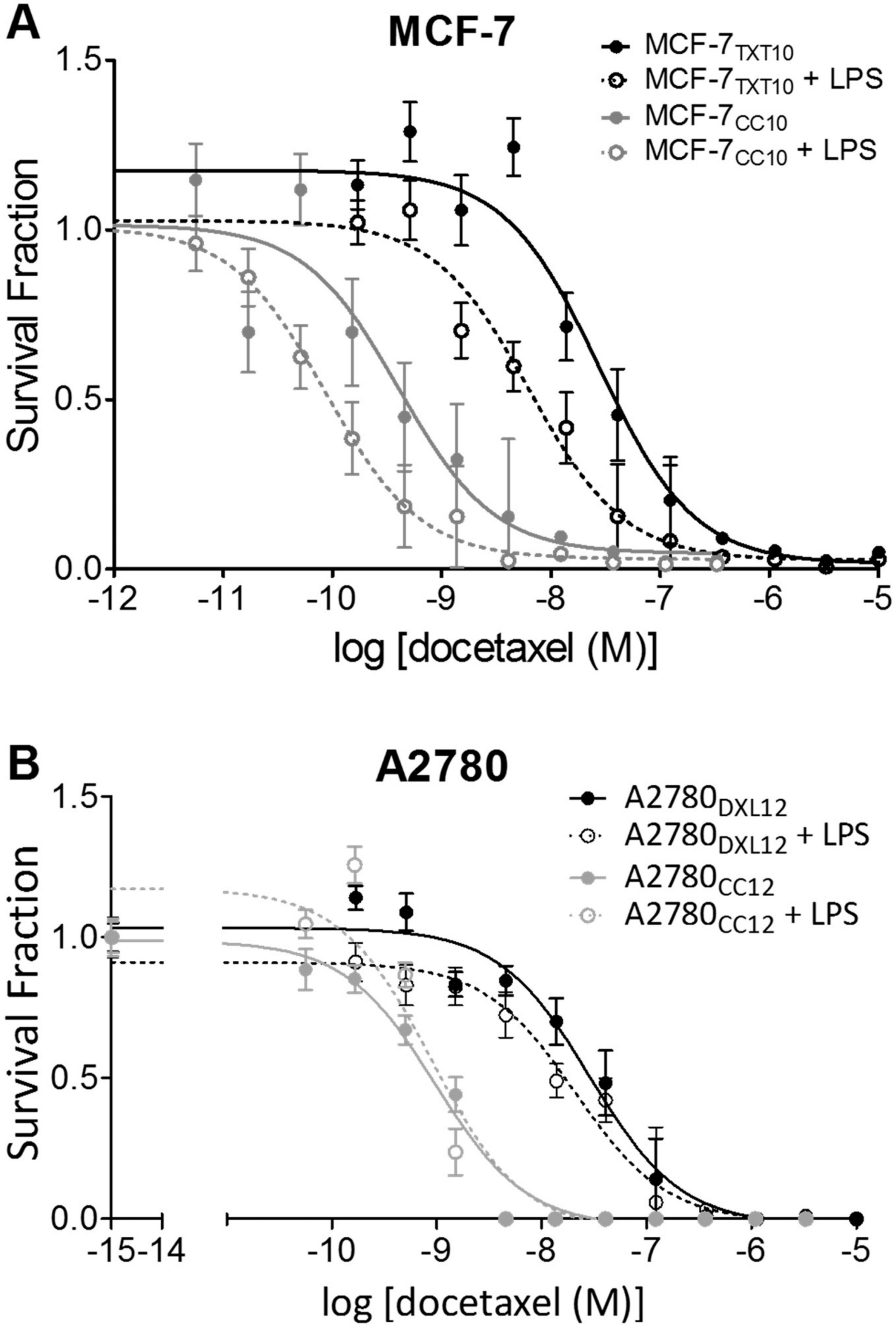
Effect of LPS exposure on tumor cell sensitivity to docetaxel. (A) Sensitivity to docetaxel was assessed using the clonogenic assay. MCF-7_CC10_ cells had an IC_50_ value for docetaxel of 0.4 nM docetaxel, and when pretreated with 10 μg/ml LPS, the value was reduced to 0.09 nM docetaxel. Likewise, MCF-7_TXT10_ cells exhibited an IC_50_ value for docetaxel of 27.6 nM docetaxel, which was reduced to 6.4 nM upon addition of 10 μg/ml LPS. Non-linear regression analysis comparing LPS-treated and LPS-absent curves revealed statistically significant differences in IC_50_ values (p<0.005). (B) Untreated and LPS-treated A2780_CC12_ cells both exhibited an IC_50_ value of 1.0 nM docetaxel. There was no significant difference in the IC_50_ values for docetaxel between LPS-treated and untreated A2780_DXL12_ cells (28.7 nM and 21.0 nM, respectively). Each data point is the mean of 12 microscopy field counts and each curve is representative of three replicate experiments, each showing a consistent trend.

The ability of LPS (but not docetaxel) to induce TNF-α release from docetaxel-resistant MCF-7_TXT10_ cells (Fig 11A) prompted us to test whether LPS could potentiate docetaxel cytotoxicity in the above docetaxel-resistant cell lines. We found that LPS increased docetaxel cytotoxicity in both MCF-7_CC10_ and MCF-7_TXT10_ cells (between 4 and 5-fold) (Fig 14A). In contrast, the sensitivity of docetaxel-resistant A2780 cells and their co-cultured control cells was unchanged upon addition of LPS (Fig 14B), consistent with their inability to manifest LPS-induced TNF-αrelease.

## Discussion

### Characterization of docetaxel-induced TNF-α release

Findings in this study suggest that several human tumor cell lines respond to docetaxel by increasing their release of TNF-α. However, the kinetics of taxane-induced TNF-α induction in human tumor cells are remarkably distinct from macrophages, as the latter requires drug concentrations in the 1 to 10 μM range, producing a maximal response within 90 minutes [29]. In contrast, the former requires low nM concentrations of taxanes (Fig 1C and 1D) and TNF-α release from tumor cells is maximal at 96 hours (Fig 1A and 1B). While much higher concentrations of paclitaxel were used in studies involving murine macrophages, it is also possible that lower drug concentrations over longer time intervals may also result in TNF-α release from macrophages. From a clinical perspective, peak plasma concentrations for docetaxel in cancer patients can be as high as 5 μM [63]. If a small fraction of circulating drug reaches cells within tumors, then the low nM concentrations of taxanes required for TNF-α induction are likely clinically relevant for cancer patient tumors.

We also observed that a variety of structurally distinct chemotherapy drugs with distinct mechanisms of action induce TNF-α release from breast and ovarian tumor cell lines with similar kinetics (Fig 2). Of the drugs tested, the taxanes appear most effective at inducing TNF-α release. While the release of TNF-α from cells after chemotherapy drug exposure could be the result of cytolysis or secondary necrosis, our findings suggest that docetaxel-induced TNF-α release in this context is not a passive process associated with lysis. Rather, our studies suggest that docetaxel-induced TNF-α release appears to involve, at least in part, the increased production of TNF-α transcripts (Fig 3). The time required for increased transcription (36 hours) suggests that chemotherapy drugs are acting through one or more intermediary products. Interestingly, aside from myeloid-based cells, non-tumor cell lines of human origin tend to be poorly responsive to taxanes in terms of TNF-α production [64,65].

### Docetaxel induces TNF-α production by a mechanism distinct from lipopolysaccharides (LPS)

Prior studies have suggested that TLR4-null macrophages exhibited lower TNF-α and nitrogen oxide (NO) production in response to paclitaxel than macrophages with wildtype TLR4 levels [30]. This suggested a role for TLR4 in paclitaxel-induced TNF-α release. The study further showed that the TLR adaptor protein MyD88 was also required, since MyD88-knockout mice did not exhibit augmented TNF-α release by paclitaxel or LPS [30]. In contrast, docetaxel-induced TNF-α release from tumor cells appears to be independent of MyD88, since we observed strong docetaxel-induced increases in soluble TNF-α levels in A2780 cells that lack detectable MyD88 expression (Fig 4B). This is in contrast to LPS-induced TNF-α production, since A2780 cells lacking MyD88 expression did not respond to LPS with increased secretion of TNF-α (Fig 11). Several prior studies have shown a lack of LPS-induced TNF-α production in MyD88-deficient cell lines [31–33,66]. Another contrast between LPS and docetaxel, is that the former induces production of TNF-α, CXCL8 and CXCL1 in MyD88-expressing MCF-7, while the latter only induced TNF-α and CXCL8 production (Fig 4).

Based on gene inactivation experiments, it is believed that membrane-bound mTNF-α is cleaved by the MMP ADAM-17, to release the soluble form of TNF-α [56] and MMP inhibitors such as Marimastat have been shown to inhibit the release of TNF-α from cells. In our study, Marimastat caused only a partial (50%) decrease in docetaxel-induced TNF-α release in A2780 cells, but not in MCF-7 cells (Fig 6). It is possible that the lack of effect of Marimastat on TNF-α release in MCF-7 cells may be due to the involvement of other Marimastat-insensitive serine proteases in the shedding of mTNF-α, as this has been demonstrated by others in alveolar macrophages [67,68]. Alternatively, inhibition by Marimastat may have been suboptimal in this system and higher concentrations could have achieved complete inhibition of mTNF-α release by LPS. Another possibility is that shedding may occur within the Golgi apparatus [69], sheltered from inhibition by Marimastat.

### Changes in cellular cytokine levels associated with the acquisition of resistance to docetaxel

Resistance to several classes of chemotherapy agents in the same cell type (multidrug resistance) has been widely studied *in vitro* and the ABC family member P-gp has been shown to induce multidrug resistance [70]. This drug transporter promotes the efflux of a variety of anti-cancer drugs [59,60,71–73]. Levels of *MDR1*, the gene that encodes P-gp, have been found to be frequently elevated in tumors that are innately resistant to chemotherapy treatment [74–76]. Its increased expression in patient tumors has also been observed in response to chemotherapy [60,74,77,78]. We have shown that selection of tumor cell lines for resistance to docetaxel is associated with increased expression of P-gp and decreased cellular accumulation of drugs (Fig 7). Treatment of docetaxel-resistant breast and ovarian tumor cells with the P-gp inhibitor Tariquidar restored cellular drug accumulation as well as drug cytotoxicity (Figs 7B and 8). While P-gp clearly contributes to docetaxel resistance in MCF-7_TXT10_ cells, it is possible that there are other contributors to resistance at lower selection doses where P-gp expression is considerably lower (MCF-7_TXT9_ cells). Aside from P-gp, whose expression continued to rise with increasing selection dose in MCF-7_TXT_ cell lines (Fig 7A), basal levels of secreted cytokines TNF-α, CXCL1, and CXCL8 also became elevated during selection for docetaxel resistance. Maximum levels of these cytokines were reached at selection dose 10 (MCF-7_TXT10_), after which they declined toward that of drug-naïve MCF-7_CC_ cells (Fig 9A, 9C and 9E). A similar trend was observed for cytokines TNF-α and CXCL1 when A2780 ovarian tumor cells were selected for docetaxel resistance (Fig 9B, 9D and 9F).

Elevated production of the cytokines CXCL8 and CXCL1 have been implicated in autocrine-based drug resistance in various tumor cell lines [24,26] and in poor clinical outcome in ovarian cancer patients [79]. CXCL8 signaling, through CXCR1 and CXCR2 promotes multidrug resistance [80,81], while CXCL1 expression, in response to taxane or anthracycline treatment [26], has been shown to cause drug-resistance through activation of CXCR2 [26,82,83] via autocrine signaling. Other groups also report an *in vivo* role for CXCL1 in drug resistance that involves paracrine signaling between tumor and myeloid cells [25].

Previous findings from our laboratory have shown that acquisition of resistance to docetaxel in MCF-7 cells was accompanied by increased TNF-α production and secretion, as well as decreased cellular levels of TNFR1 [23]. Blockade of TNFR2 in the resistant cells restored sensitivity to docetaxel by roughly 2-fold [23]. It is unclear whether these alterations in TNF-α signaling contribute to drug resistance through modulations of P-gp expression or activity. Exogenously added sTNF-α has been shown to stimulate P-gp expression and activity in a variety of cell lines [84–87]; however, we observed that exposure of MCF-7 cells to sTNF-α failed to promote detectable increases in P-gp at the protein level after treatment for 96 hours (see S1 Fig). At any rate, it is possible that autocrine signaling by mTNF-αmay be one of many confounding factors, given studies showing that membrane-bound and soluble forms of TNF-α, expressed by tumor cells, have opposing effects on tumor-associated myeloid cells [19]. It is unknown whether mTNF-α levels are increased in the drug-selected cell lines used in this study.

If one or more of the above inflammatory cytokines promote P-gp expression in MCF-7 cells, then it would follow that this support would be lost beyond selection dose 10 (15 nM docetaxel), since cellular levels of these cytokines fall considerably at higher selection doses (Fig 9). Yet, we observe P-gp expression to increase further. Likely, this involves another mechanism to increase P-gp expression, as above selection dose 10, we detected a regional amplification on chromosome 7 (7q21) resulting in an increased P-gp (*MDR1*) gene copy number [58]. This amplification results in highly increased P-gp expression, which may no longer be driven by elevated cytokine production. Recent studies also support a role for TNF-α as a potent mutagen [19,88], since treatment of cultured cells with sTNF-α was found to cause DNA damage comparable to that of ionizing radiation [88]. This, in turn, caused gene amplifications, mutations, micronuclei formation, and greater chromosomal instability [88], which could have led to the chromosomal amplifications we observed in MCF-7_TXT11_ and MCF-7_TXT12_ cells.

In our study, we observed that despite the increased basal output of TNF-α from both breast and ovarian tumor cell lines during acquisition of docetaxel resistance, there was a diminished ability to further increase TNF-α production in response to docetaxel (Fig 11). In contrast, LPS retained its ability to induce TNF-α production in MCF-7_TXT10_ cells. The ability of Tariquidar to re-establish both docetaxel accumulation and cytotoxicity in MCF-7_TXT10_ and A2780_DXL12_ cells suggests that docetaxel’s ability to induce TNF-α release requires drug entry into tumor cells. This is unlike LPS, which promotes TNF-α production by extracellular binding to TLR4. Consistent with previous reports that MyD88 is an essential cellular component for LPS-induced inflammatory cytokine production, elevated TNF-α production in response to LPS treatment did not occur in A2780_DXL_ cells, which do not express detectable levels of MyD88 (Fig 12).

### Mechanistic insights into docetaxel-induced TNF-α production

Some groups have reported that paclitaxel induces TNF-α release through the activation of TLR4, via a direct interaction between paclitaxel and the murine extracellular TLR4 adaptor protein MD-2 at the cell surface of macrophages and HEK293 cells [34,64]. This appears to be the accepted mechanism, by which taxanes promote TNF-α production and release in human macrophages [29,30] as well as tumor cells [33,43,89], despite the lack of rigorous studies employing tumor cells. Although our findings do not discount a role for TLR4 in mediating taxane-induced TNF-α production in all tumor cell types, they do suggest a distinct mechanism involving drug entry into tumor cells, with or without an indirect activation of TLR4.

Our observation that a variety of structurally unrelated chemotherapy agents can induce TNF-α release suggests that a variety of pathways promote this phenomenon. A recent review also questions whether the functional effects of paclitaxel on TNF-αsecretion are mediated through physical binding to TLR4 [90]. One possibility is that TLR4 activation by chemotherapy drugs involves the death-dependent release of DAMPs, a subset of alarmins, some of which can activate TLR4 and subsequent cytokine production. For example, studies have demonstrated the release of the DAMP HMGB1 after treatment with a variety of chemotherapy agents, including docetaxel [14]. HMGB1 among other alarmins has been shown to activate TLR4 after its release from necrotic cells [12]. The release of DAMPs in response to docetaxel could require the drug’s uptake into cells. Drug-induced TNF-α production may also involve heat shock proteins (HSP's), as geldanamycin, a specific inhibitor for the Hsp90 family, abrogated the expression of TNF-α in macrophages treated with paclitaxel or LPS [91]. Interestingly, geldanamycin did not block microtubule stabilization by paclitaxel suggesting a mechanism independent of the drug’s effect on microtubules [91]. The authors of the study further suggest that paclitaxel may bind to Hsp90, mediating macrophage activation [91].

Kawasaki et al. demonstrated that paclitaxel's ability to mimic the effects of LPS occurs only in murine-derived cell lines, as the expression of recombinant *murine* MD-2 in combination with either human or murine TLR4 is a requirement for inflammatory cytokine expression [34]. Resman et al. reported an *inhibitory* effect of docetaxel on LPS-induced TLR4 signaling, through binding of MD-2 in human embryonic kidney (HEK293) cells [65]. There are many potential explanations for the lack of taxane-induced inflammatory response in human tissue. As we've seen in this study, a response involving TNF-α release is clearly dose-dependent (Fig 1). Furthermore, the optimal dose likely differs greatly between tissues, as most studies reporting this phenomenon in macrophages or other non-malignant cell lines involve treatment with very high taxane concentrations roughly three orders of magnitude greater than those used here. However, it is interesting to note that LPS-RS (Lipid IVA) suppressed TNF-α production in breast tumor cells treated with LPS, but in the same cell line potentiated TNF-α release in the presence of docetaxel (Fig 5A). This is consistent with studies demonstrating a pro-inflammatory role for lipid IVA, *exclusively* in murine-derived cells [48]. Thus, expression of some confounding factor associated with the *murine* form of MD-2, yet induced by docetaxel, may confer responsiveness to penta-acylated forms of LPS. In addition, interfering with the intracellular signaling cascade triggered by extracellular TLR4 ligation was shown in our study to be insufficient for preventing docetaxel-induced TNF-α release from MB-231 cells (Fig 5B). The absence of an inhibitory effect of TAK-242 on docetaxel-induced TNF-α release at 1 μg/ml, a concentration that completely inhibits LPS-induced TNF-α release, suggests that TLR4 is not an important contributor to TNF-α release by docetaxel in the MDA-MB-231 cell line. Nevertheless, further studies are clearly warranted for a better understanding of the mechanisms at play.

#### Effects of LPS pretreatment on docetaxel cytotoxicity

In this study, we found that pretreatment of drug-naive and docetaxel-resistant MCF-7 cells with LPS significantly increased sensitivity to docetaxel, but this effect was absent in MyD88-deficient A2780 and A2780-derived cell lines (Fig 14). Although it is unclear how LPS elicits this increased sensitization to docetaxel in MCF-7 cells, it arguably does not involve changes in P-gp activity or expression, since LPS also caused sensitization to docetaxel in drug-naive cells that lack detectable P-gp levels. It is interesting to note that the sensitizing effect of LPS in MCF-7 cells was accompanied by a cellular response to LPS involving increased cytokine production (TNF-α, CXCL8, and CXCL1). Similarly, the inability of LPS to increase sensitivity to docetaxel in MyD88-deficient A2780 cells was associated with its inability to promote increased production of these cytokines (Figs 4 and 11). This suggests that the biochemical pathways that are activated by LPS, resulting in inflammatory cytokine release, are closely related to those responsible for the increased sensitization to docetaxel. Given the reported ability of TNF-α to sensitize tumor cells to taxanes [23,92], the docetaxel-sensitizing effects observed here (Fig 14) may likewise be provoked through LPS's ability to induce TNF-α production and subsequent death-associated autocrine signaling.

It should be noted that the effects of LPS exposure on cellular sensitivity to docetaxel have been studied by other groups. In contrast to our observations, one study shows that ligation of TLR4 with LPS induced increased resistance to the growth inhibitory effects of docetaxel on PC-3 prostate tumor cells [93], and other groups have shown that TLR4 activation is associated with increased resistance to taxanes in SKOV-3 [31]. Consistent with our results, however, LPS was found to have no effect in A2780 [31]. Thus it would appear that depending on the particular tumor cell line in question, LPS exposure can either increase, decrease or have no effect on sensitivity to docetaxel.

## Conclusion

The pathogen recognition receptor TLR4 is undoubtedly involved in the induction of inflammatory cytokines and is expressed in a variety of cell types, most notably immune cells [94] and also tumor cells as our data illustrates. The activation of this receptor can occur during exposure to the pathogen-associated molecular pattern LPS, and also in response to chemotherapy drugs of the taxane family [30,43]. Although taxanes can induce the production of inflammatory cytokines through activation of TLR4 in both murine macrophages [95], and certain tumor cell types [43], we have observed that this response can occur independently of TLR4 in the MDA-MB-231 breast tumor cell line. We also demonstrate that docetaxel-induced cytokine production in the ovarian A2780 and breast MCF-7 cell lines requires the cellular accumulation of drug and thus suggests, contrary to popular belief, that it does not involve the direct interaction of drug with TLR4 at the cell surface in these cell lines. Regardless of its mode of activation, TLR4 is an ideal target for the activation of inflammatory pathways and its activation has become a promising strategy for therapeutic intervention and the development of vaccine adjuvants [96]. Although LPS is the most studied ligand for TLR4, its known toxicity in humans limits its clinical use as a vaccine adjuvant [96]. However, much of the LPS structure that is responsible for its toxicity has proven unnecessary for TLR4 activation [97]. This knowledge has prompted the study of less toxic agonists [98,99], which are currently used in a variety of adjuvant formulations [96]. In light of our novel reports in MCF-7, these clinically tested agonists may be worthy of assessment for their ability to augment chemotherapy response, as they may also be able to simultaneously improve immune recognition of the tumor during chemotherapy treatment of patients.

## Supporting information

S1 Fig Levels of P-gp protein expression in breast tumor cells after supplementation with exogenous recombinant TNF-α. The medium of MCF-7_CC10_ cells was supplemented with 10 ng/ml TNF-α twice over 96 hours (second treatment at 48 hours), after which cell surface P-gp protein expression was assessed in treated and untreated cells by flow cytometry. This was then compared to P-gp expression in untreated MCF-7_TXT10_ cells using the same approach. An ANOVA with Tukey post-test was then used to assess the significance of differences in P-gp expression among the samples (***p = 0.0002).
(TIF)

S1 File Raw data for all manuscript figures. (PDF)

## References

[1] Centers for Disease Control and Prevention n.d. http://www.cdc.gov/cancer/international/statistics.htm (accessed January 7, 2016).

[2] GLOBOCAN 2012: Estimated Cancer Incidence, Mortality and Prevalence Worldwide in 2012 n.d. http://globocan.iarc.fr/Pages/fact_sheets_cancer.aspx (accessed January 7, 2016).

[3] Smith I, Chua S. Medical treatment of early breast cancer. I: adjuvant treatment. BMJ 2006;332:34–7. doi: 10.1136/bmj.332.7532.34 16399737 10.1136/bmj.332.7532.34 PMC1325133

[4] Fisher B, Bryant J, Wolmark N, Mamounas E, Brown a, Fisher ER, et al Effect of preoperative chemotherapy on the outcome of women with operable breast cancer. J Clin Oncol 1998;16:2672–85. doi: 10.1200/JCO.1998.16.8.2672 9704717 10.1200/JCO.1998.16.8.2672

[5] Eltahir A, Heys SD, Hutcheon AW, Sarkar TK, Smith I, Walker LG, et al Treatment of large and locally advanced breast cancers using neoadjuvant chemotherapy. Am J Surg 1998;175:127–32. S0002961097002791 [pii]. 9515529 10.1016/s0002-9610(97)00279-1

[6] Smith I, Chua S. Medical treatment of early breast cancer. IV: neoadjuvant treatment. BMJ 2006;332:223–4. doi: 10.1136/bmj.332.7535.223 16439402 10.1136/bmj.332.7535.223 PMC1352059

[7] Crown J, O’Leary M, Ooi W-S. Docetaxel and paclitaxel in the treatment of breast cancer: a review of clinical experience. Oncologist 2004;9 Suppl 2:24–32. 15161988 10.1634/theoncologist.9-suppl_2-24

[8] Martin M, Pienkowski T, Mackey J, Pawlicki M, Guastalla J-P, Weaver C, et al Adjuvant docetaxel for node-positive breast cancer. N Engl J Med 2005;352:2302–13. doi: 10.1056/NEJMoa043681 15930421 10.1056/NEJMoa043681

[9] Martín M, Seguí M a, Antón A, Ruiz A, Ramos M, Adrover E, et al Adjuvant docetaxel for high-risk, node-negative breast cancer. N Engl J Med 2010;363:2200–10. doi: 10.1056/NEJMoa0910320 21121833 10.1056/NEJMoa0910320

[10] Markauskas A, Mogensen O, dePont Christensen R, Jensen PT. Primary Surgery or Interval Debulking for Advanced Epithelial Ovarian Cancer. Int J Gynecol Cancer 2014;24:1420–8. doi: 10.1097/IGC.0000000000000241 25180461 10.1097/IGC.0000000000000241

[11] Agarwal R, Kaye SB. Ovarian cancer: strategies for overcoming resistance to chemotherapy. Nat Rev Cancer 2003;3:502–16. doi: 10.1038/nrc1123 12835670 10.1038/nrc1123

[12] Apetoh L, Ghiringhelli F, Tesniere A, Criollo A, Ortiz C, Lidereau R, et al The interaction between HMGB1 and TLR4 dictates the outcome of anticancer chemotherapy and radiotherapy. Immunol Rev 2007;220:47–59. doi: 10.1111/j.1600-065X.2007.00573.x 17979839 10.1111/j.1600-065X.2007.00573.x

[13] Bianchi ME. DAMPs, PAMPs and alarmins: all we need to know about danger. J Leukoc Biol 2007;81:1–5. doi: 10.1189/jlb.0306164 17032697 10.1189/jlb.0306164

[14] Buoncervello M, Borghi P, Romagnoli G, Spadaro F, Belardelli F, Toschi E, et al Apicidin and docetaxel combination treatment drives CTCFL expression and HMGB1 release acting as potential antitumor immune response inducers in metastatic breast cancer cells. Neoplasia 2012;14:855–67. doi: 10.1593/neo.121020 23019417 10.1593/neo.121020 PMC3459281

[15] Trevejo JM, Marino MW, Philpott N, Josien R, Richards EC, Elkon KB, et al TNF-alpha -dependent maturation of local dendritic cells is critical for activating the adaptive immune response to virus infection. Proc Natl Acad Sci U S A 2001;98:12162–7. doi: 10.1073/pnas.211423598 11593031 10.1073/pnas.211423598 PMC59785

[16] Sedgwick JD, Riminton DS, Cyster JG, K??rner H. Tumor necrosis factor: A master-regulator of leukocyte movement. Immunol Today 2000;21:110–3. doi: 10.1016/S0167-5699(99)01573-X 10689296 10.1016/s0167-5699(99)01573-x

[17] Bradley JR. TNF-mediated inflammatory disease. J Pathol 2008;214:149–60. doi: 10.1002/path.2287 18161752 10.1002/path.2287

[18] Parameswaran N, Patial S. Tumor necrosis factor-α signaling in macrophages. Crit Rev Eukaryot Gene Expr 2010;20:87–103. 21133840 10.1615/critreveukargeneexpr.v20.i2.10 PMC3066460

[19] Ardestani S, Li B, Deskins DL, Wu H, Massion PP, Young PP. Membrane versus soluble isoforms of TNF-?? exert opposing effects on tumor growth and survival of tumor-associated myeloid cells. Cancer Res 2013;73:3938–50. doi: 10.1158/0008-5472.CAN-13-0002 23704210 10.1158/0008-5472.CAN-13-0002 PMC3702680

[20] Li B, Vincent A, Cates J, Brantley-Sieders DM, Polk DB, Young PP. Low levels of tumor necrosis factor alpha increase tumor growth by inducing an endothelial phenotype of monocytes recruited to the tumor site. Cancer Res 2009;69:338–48. doi: 10.1158/0008-5472.CAN-08-1565 19118019 10.1158/0008-5472.CAN-08-1565 PMC2651676

[21] Modur V, Zimmerman GA, Prescott SM, McIntyre TM. Endothelial cell inflammatory responses to tumor necrosis factor alpha. Ceramide-dependent and -independent mitogen-activated protein kinase cascades. J Biol Chem 1996;271:13094–102. doi: 10.1074/JBC.271.22.13094 8662702 10.1074/jbc.271.22.13094

[22] Ma TY, Iwamoto GK, Hoa NT, Akotia V, Pedram A, Boivin M a, et al TNF-alpha-induced increase in intestinal epithelial tight junction permeability requires NF-kappa B activation. Am J Physiol Gastrointest Liver Physiol 2004;286:G367–76. doi: 10.1152/ajpgi.00173.2003 14766535 10.1152/ajpgi.00173.2003

[23] Sprowl JA, Reed K, Armstrong SR, Lanner C, Guo B, Kalatskaya I, et al Alterations in tumor necrosis factor signaling pathways are associated with cytotoxicity and resistance to taxanes: a study in isogenic resistant tumor cells. Breast Cancer Res 2012;14:R2 doi: 10.1186/bcr3083 22225778 10.1186/bcr3083 PMC3496117

[24] Wang Y, Qu Y, Niu XL, Sun WJ, Zhang XL, Li LZ. Autocrine production of interleukin-8 confers cisplatin and paclitaxel resistance in ovarian cancer cells. Cytokine 2011;56:365–75. doi: 10.1016/j.cyto.2011.06.005 21742513 10.1016/j.cyto.2011.06.005

[25] Acharyya S, Oskarsson T, Vanharanta S, Malladi S, Kim J, Morris PG, et al A CXCL1 paracrine network links cancer chemoresistance and metastasis. Cell 2012;150:165–78. doi: 10.1016/j.cell.2012.04.042 22770218 10.1016/j.cell.2012.04.042 PMC3528019

[26] Sharma B, Nawandar DM, Nannuru KC, Varney ML, Singh RK. Targeting CXCR2 enhances chemotherapeutic response, inhibits mammary tumor growth, angiogenesis, and lung metastasis. Mol Cancer Ther 2013;12:799–808. doi: 10.1158/1535-7163.MCT-12-0529 23468530 10.1158/1535-7163.MCT-12-0529 PMC3653628

[27] Lu L, Li ZJ, Li LF, Wu WKK, Shen J, Zhang L, et al Vascular-targeted TNFα improves tumor blood vessel function and enhances antitumor immunity and chemotherapy in colorectal cancer. J Control Release 2015;210:134–46. doi: 10.1016/j.jconrel.2015.05.282 26003042 10.1016/j.jconrel.2015.05.282

[28] Trédan O, Galmarini CM, Patel K, Tannock IF. Drug resistance and the solid tumor microenvironment. J Natl Cancer Inst 2007;99:1441–54. doi: 10.1093/jnci/djm135 17895480 10.1093/jnci/djm135

[29] Bogdan C, Ding A. Taxol, a microtubule-stabilizing antineoplastic agent, induces expression of tumor necrosis factor alpha and interleukin-1 in macrophages. J Leukoc Biol 1992;52:119–21. 1353517 10.1002/jlb.52.1.119

[30] Byrd-Leifer CA, Block EF, Takeda K, Akira S, Ding A. The role of MyD88 and TLR4 in the LPS-mimetic activity of Taxol. Eur J Immunol 2001;31:2448–57. doi: 10.1002/1521-4141(200108)31:8<2448::AID-IMMU2448>3.0.CO;2-N 11500829 10.1002/1521-4141(200108)31:8<2448::aid-immu2448>3.0.co;2-n

[31] Szajnik M, Szczepanski MJ, Czystowska M, Elishaev E, Mandapathil M, Nowak-Markwitz E, et al TLR4 signaling induced by lipopolysaccharide or paclitaxel regulates tumor survival and chemoresistance in ovarian cancer. Oncogene 2009;28:4353–63. doi: 10.1038/onc.2009.289 19826413 10.1038/onc.2009.289 PMC2794996

[32] Kelly MG, Alvero AB, Chen R, Silasi D-A, Abrahams VM, Chan S, et al TLR-4 signaling promotes tumor growth and paclitaxel chemoresistance in ovarian cancer. Cancer Res 2006;66:3859–68. doi: 10.1158/0008-5472.CAN-05-3948 16585214 10.1158/0008-5472.CAN-05-3948

[33] Wang AC, Ma YB, Wu FX, Ma ZF, Liu NF, Gao R, et al TLR4 induces tumor growth and inhibits paclitaxel activity in MyD88-positive human ovarian carcinoma in vitro. Oncol Lett 2014;7:871–7. doi: 10.3892/ol.2013.1759 24527095 10.3892/ol.2013.1759 PMC3919821

[34] Kawasaki K, Akashi S, Shimazu R, Yoshida T, Miyake K, Nishijima M. Mouse Toll-like Receptor 4·MD-2 Complex Mediates Lipopolysaccharide-mimetic Signal Transduction by Taxol. J Biol Chem 2000;275:2251–4. doi: 10.1074/jbc.275.4.2251 10644670 10.1074/jbc.275.4.2251

[35] Guo B, Villeneuve DJ, Hembruff SL, Kirwan AF, Blais DE, Bonin M, et al Cross-resistance studies of isogenic drug-resistant breast tumor cell lines support recent clinical evidence suggesting that sensitivity to paclitaxel may be strongly compromised by prior doxorubicin exposure. Breast Cancer Res Treat 2004;85:31–51. doi: 10.1023/B:BREA.0000021046.29834.12 15039596 10.1023/B:BREA.0000021046.29834.12

[36] Armstrong SR, Narendrula R, Guo B, Parissenti AM, McCallum KL, Cull S, et al Distinct genetic alterations occur in ovarian tumor cells selected for combined resistance to carboplatin and docetaxel. J Ovarian Res 2012;5:40 doi: 10.1186/1757-2215-5-40 23194409 10.1186/1757-2215-5-40 PMC3541348

[37] Bogdan C, Ding A. Taxol, a microtubule-stabilizing antineoplastic agent, induces expression of tumor necrosis factor alpha and interleukin-1 in macrophages. J Leukoc Biol 1992;52:119–21. 1353517 10.1002/jlb.52.1.119

[38] Parker M. M., Parrillo JE. Septic Shock. Hemodynamics and pathogenesis. JAMA 1983:3324–7. 6196497

[39] Tracey KJ, Beutler B, Lowry SF, Merryweather J, Wolpe S, Milsark IW, et al Shock and tissue injury induced by recombinant human cachectin. Science 1986;234:470–4. 3764421 10.1126/science.3764421

[40] Chow JC, Young DW, Golenbock DT, Christ WJ, Gusovsky F. Toll-like Receptor-4 Mediates Lipopolysaccharide-induced Signal Transduction. J Biol Chem 1999;274:10689–92. doi: 10.1074/jbc.274.16.10689 10196138 10.1074/jbc.274.16.10689

[41] Schletter J, Heine H, Ulmer AJ, Rietschel ET. Molecular mechanisms of endotoxin activity. Arch Microbiol 1995;164:383–9. doi: 10.1007/BF02529735 8588739 10.1007/BF02529735

[42] Yang RB, Mark MR, Gray A, Huang A, Xie MH, Zhang M, et al Toll-like receptor-2 mediates lipopolysaccharide-induced cellular signalling. Nature 1998;395:284–8. doi: 10.1038/26239 9751057 10.1038/26239

[43] Szajnik M, Szczepanski MJ, Czystowska M, Elishaev E, Mandapathil M, Nowak-Markwitz E, et al TLR4 signaling induced by lipopolysaccharide or paclitaxel regulates tumor survival and chemoresistance in ovarian cancer. Oncogene 2009;28:4353–63. doi: 10.1038/onc.2009.289 19826413 10.1038/onc.2009.289 PMC2794996

[44] Wu Z, Wang S, Wu I, Mata M, Fink DJ. Activation of TLR-4 to produce tumour necrosis factor-α in neuropathic pain caused by paclitaxel. Eur J Pain 2015;19:889–98. doi: 10.1002/ejp.613 25388329 10.1002/ejp.613

[45] Vitiello M, D’Isanto M, Galdiero M, Raieta K, Tortora A, Rotondo P, et al Interleukin-8 production by THP-1 cells stimulated by Salmonella enterica serovar Typhimurium porins is mediated by AP-1, NF-kappaB and MAPK pathways. Cytokine 2004;27:15–24. doi: 10.1016/j.cyto.2004.03.010 15207247 10.1016/j.cyto.2004.03.010

[46] De Filippo K, Dudeck A, Hasenberg M, Nye E, Van Rooijen N, Hartmann K, et al Mast cell and macrophage chemokines CXCL1/CXCL2 control the early stage of neutrophil recruitment during tissue inflammation. Blood 2013;121:4930–7. doi: 10.1182/blood-2013-02-486217 23645836 10.1182/blood-2013-02-486217

[47] Reife RA, Darveau RP, Coats SR, Pham T-TT, Bainbridge BW. MD-2 Mediates the Ability of Tetra-Acylated MD-2 Mediates the Ability of Tetra-Acylated and Penta-Acylated Lipopolysaccharides to Antagonize Escherichia coli Lipopolysaccharide at the TLR4 Signaling Complex. J Immunol Ref 2005;175:4490–8. doi: 10.4049/jimmunol.175.7.4490 10.4049/jimmunol.175.7.4490 16177092

[48] Golenbock DT, Hampton RY, Qureshi N, Takayama K, Raetz CR. Lipid A-like molecules that antagonize the effects of endotoxins on human monocytes. J Biol Chem 1991;266:19490–8. 1918061

[49] Visintin A, Halmen K, Latz E, Monks B, Golenbock D. Pharmacological inhibition of endotoxin responses is achieved by targeting the TLR4 coreceptor, MD-2. Open Access Artic 2005. 10.4049/jimmunol.175.10.6465 16272300

[50] Saitoh S, Akashi S, Yamada T, Tanimura N, Kobayashi M, Konno K, et al Lipid A antagonist, lipid IVa, is distinct from lipid A in interaction with Toll-like receptor 4 (TLR4)-MD-2 and ligand-induced TLR4 oligomerization. Int Immunol 2004;16:961–9. doi: 10.1093/intimm/dxh097 15184344 10.1093/intimm/dxh097

[51] Matsunaga N, Tsuchimori N, Matsumoto T, Ii M. TAK-242 (Resatorvid), a Small-Molecule Inhibitor of Toll-Like Receptor (TLR) 4 Signaling, Binds Selectively to TLR4 and Interferes with Interactions between TLR4 and Its Adaptor Molecules. Mol Pharmacol 2011;79:34–41. doi: 10.1124/mol.110.068064 20881006 10.1124/mol.110.068064

[52] Good DW, George T, Watts BA. Toll-like receptor 2 is required for LPS-induced Toll-like receptor 4 signaling and inhibition of ion transport in renal thick ascending limb. J Biol Chem 2012;287:20208–20. doi: 10.1074/jbc.M111.336255 22523073 10.1074/jbc.M111.336255 PMC3370203

[53] Wang Y-C, Zhou Y, Fang H, Lin S, Wang P-F, Xiong R-P, et al Toll-like receptor 2/4 heterodimer mediates inflammatory injury in intracerebral hemorrhage. Ann Neurol 2014;75:876–89. doi: 10.1002/ana.24159 24752976 10.1002/ana.24159

[54] Stanley AC, Lacy P. Pathways for cytokine secretion. Physiology (Bethesda) 2010;25:218–29. doi: 10.1152/physiol.00017.2010 20699468 10.1152/physiol.00017.2010

[55] Raabe T, Bukrinsky M, Currie RA. Relative contribution of transcription and translation to the induction of tumor necrosis factor-alpha by lipopolysaccharide. J Biol Chem 1998;273:974–80. 9422758 10.1074/jbc.273.2.974

[56] Black RA, Rauch CT, Kozlosky CJ, Peschon JJ, Slack JL, Wolfson MF, et al A metalloproteinase disintegrin that releases tumour-necrosis factor-alpha from cells. Nature 1997;385:729–33. doi: 10.1038/385729a0 9034190 10.1038/385729a0

[57] Rasmussen HS, McCann PP. Matrix metalloproteinase inhibition as a novel anticancer strategy: A review with special focus on Batimastat and Marimastat. Pharmacol Ther 1997;75:69–75. doi: 10.1016/S0163-7258(97)00023-5 9364582 10.1016/s0163-7258(97)00023-5

[58] Reed K, Hembruff SL, Laberge ML, Villeneuve DJ, Côté GB, Parissenti AM. Hypermethylation of the ABCB1 downstream gene promoter accompanies ABCB1 gene amplification and increased expression in docetaxel-resistant MCF-7 breast tumor cells. Epigenetics 2008;3:270–80. 6868 [pii]. 19001875 10.4161/epi.3.5.6868

[59] Shirakawa K, Takara K, Tanigawara Y, Aoyama N, Kasuga M, Komada F, et al Interaction of docetaxel (“Taxotere”) with human P-glycoprotein. Jpn J Cancer Res 1999;90:1380–6. S0910505000870933 [pii]. 10665657 10.1111/j.1349-7006.1999.tb00723.x PMC5926029

[60] Nobili S, Landini I, Giglioni B, Mini E. Pharmacological strategies for overcoming multidrug resistance. Curr Drug Targets 2006;7:861–79. doi: 10.2174/138945006777709593 16842217 10.2174/138945006777709593

[61] Manna SK, Ramesh GT. Interleukin-8 induces nuclear transcription factor-kappaB through a TRAF6-dependent pathway. J Biol Chem 2005;280:7010–21. doi: 10.1074/jbc.M410994200 15591054 10.1074/jbc.M410994200 PMC2740382

[62] Jeyaseelan S, Cai S, Batra S, Lira SA, Kolls JK. CXCL1 Regulates Pulmonary Host Defense CXCL1 Regulates Pulmonary Host Defense to Klebsiella Infection via CXCL2, CXCL5, NF-kB, and MAPKs. J Immunol J Immunol 2010;185:6214–25. doi: 10.4049/jimmunol.0903843 20937845 10.4049/jimmunol.0903843 PMC2974054

[63] Baker SD, Zhao M, Lee CKK, Verweij J, Zabelina Y, Brahmer JR, et al Comparative pharmacokinetics of weekly and every-three-weeks docetaxel. Clin Cancer Res 2004;10:1976–83. doi: 10.1158/1078-0432.ccr-0842-03 15041715 10.1158/1078-0432.ccr-0842-03

[64] Zimmer SM, Liu J, Clayton JL, Stephens DS, Snyder JP. Paclitaxel binding to human and murine MD-2. J Biol Chem 2008;283:27916–26. doi: 10.1074/jbc.M802826200 18650420 10.1074/jbc.M802826200 PMC2562052

[65] Resman N, Gradišar H, Vašl J, Keber MM, Pristovšek P, Jerala R. Taxanes inhibit human TLR4 signaling by binding to MD-2. FEBS Lett 2008;582:3929–34. doi: 10.1016/j.febslet.2008.10.037 18977229 10.1016/j.febslet.2008.10.037

[66] Kawai T, Takeuchi O, Fujita T, Inoue J -i., Muhlradt PF, Sato S, et al Lipopolysaccharide Stimulates the MyD88-Independent Pathway and Results in Activation of IFN-Regulatory Factor 3 and the Expression of a Subset of Lipopolysaccharide-Inducible Genes. J Immunol 2001;167:5887–94. doi: 10.4049/jimmunol.167.10.5887 11698465 10.4049/jimmunol.167.10.5887

[67] Armstrong L, Godinho SIH, Uppington KM, Whittington HA, Millar AB. Contribution of TNF-α converting enzyme and proteinase-3 to TNF-α processing in human alveolar macrophages. Am J Respir Cell Mol Biol 2006;34:219–25. doi: 10.1165/rcmb.2005-0087OC 16210695 10.1165/rcmb.2005-0087OC

[68] Niehörster M, Tiegs G, Schade UF, Wendel A. In vivo evidence for protease-catalysed mechanism providing bioactive tumor necrosis factor alpha. Biochem Pharmacol 1990;40:1601–3. 2222515 10.1016/0006-2952(90)90461-s

[69] Shurety W, Merino-Trigo A, Brown D, Hume DA, Stow JL. Localization and post-Golgi trafficking of tumor necrosis factor-alpha in macrophages. J Interferon Cytokine Res 2000;20:427–38. doi: 10.1089/107999000312379 10805378 10.1089/107999000312379

[70] Tunggal JK, Melo T, Ballinger JR, Tannock IF. The influence of expression of P-glycoprotein on the penetration of anticancer drugs through multicellular layers. Int J Cancer 2000;86:101–7. doi: 10.1002/(SICI)1097-0215(20000401)86:1<101::AID-IJC16>3.0.CO;2-I 10728602 10.1002/(sici)1097-0215(20000401)86:1<101::aid-ijc16>3.0.co;2-i

[71] Biedler JL, Riehm H. Cellular resistance to actinomycin D in Chinese hamster cells in vitro: cross-resistance, radioautographic, and cytogenetic studies. Cancer Res 1970;30:1174–84. 5533992

[72] Mahon FX, Belloc F, Lagarde V, Chollet C, Moreau-Gaudry F, Reiffers J, et al MDR1 gene overexpression confers resistance to imatinib mesylate in leukemia cell line models. Blood 2003;101:2368–73. doi: 10.1182/blood.V101.6.2368 12609962 10.1182/blood.V101.6.2368

[73] Erba E, Bergamaschi D, Bassano L, Ronzoni S, Di Liberti G, Muradore I, et al Isolation and characterization of an IGROV-1 human ovarian cancer cell line made resistant to Ecteinascidin-743 (ET-743). Br J Cancer 2000;82:1732–9. doi: 10.1054/bjoc.2000.1224 10817511 10.1054/bjoc.2000.1224 PMC2374505

[74] Goldstein LJ, Galski H, Fojo a, Willingham M, Lai SL, Gazdar a, et al Expression of a multidrug resistance gene in human cancers. J Natl Cancer Inst 1989;81:116–24. 2562856 10.1093/jnci/81.2.116

[75] Miller TP, Grogan TM, Dalton WS, Spier CM, Scheper RJ, Salmon SE. P-glycoprotein expression in malignant lymphoma and reversal of clinical drug resistance with chemotherapy plus high-dose verapamil. J Clin Oncol 1991;9:17–24. doi: 10.1200/JCO.1991.9.1.17 1670642 10.1200/JCO.1991.9.1.17

[76] Taylor CW, Dalton WS, Mosley K, Dorr RT, Salmon SE. Combination chemotherapy with cyclophosphamide, vincristine, adriamycin, and dexamethasone (CVAD) plus oral quinine and verapamil in patients with advanced breast cancer. Breast Cancer Res Treat 1997;42:7–14. doi: 10.1023/A:1005716214718 9116320 10.1023/a:1005716214718

[77] Trock BJ, Leonessa F, Clarke R. Multidrug resistance in breast cancer: a meta-analysis of MDR1/gp170 expression and its possible functional significance. J Natl Cancer Inst 1997;89:917–31. 9214671 10.1093/jnci/89.13.917

[78] Nobili S, Napoli C, Puccini B, Landini I, Perrone G, Brugia M, et al Identification of pharmacogenomic markers of clinical efficacy in a dose-dense therapy regimen (R-CHOP14) in diffuse large B-cell lymphoma. Leuk Lymphoma 2014:1–8. doi: 10.3109/10428194.2013.866665 24289107 10.3109/10428194.2013.866665

[79] Merritt WM, Lin YG, Spannuth WA, Fletcher MS, Kamat AA, Han LY, et al Effect of interleukin-8 gene silencing with liposome-encapsulated small interfering RNA on ovarian cancer cell growth. J Natl Cancer Inst 2008;100:359–72. doi: 10.1093/jnci/djn024 18314475 10.1093/jnci/djn024 PMC2770251

[80] Shi Z, Yang W-M, Chen L-P, Yang D-H, Zhou Q, Zhu J, et al Enhanced chemosensitization in multidrug-resistant human breast cancer cells by inhibition of IL-6 and IL-8 production. Breast Cancer Res Treat 2012;135:737–47. doi: 10.1007/s10549-012-2196-0 22923236 10.1007/s10549-012-2196-0

[81] Waugh DJJ, Wilson C. The interleukin-8 pathway in cancer. Clin Cancer Res 2008;14:6735–41. doi: 10.1158/1078-0432.CCR-07-4843 18980965 10.1158/1078-0432.CCR-07-4843

[82] Brat DJ, Bellail AC, Van Meir EG. The role of interleukin-8 and its receptors in gliomagenesis and tumoral angiogenesis. Neuro Oncol 2005;7:122–33. doi: 10.1215/S1152851704001061 15831231 10.1215/S1152851704001061 PMC1871893

[83] Bolitho C, Hahn MA, Baxter RC, Marsh DJ. The chemokine CXCL1 induces proliferation in epithelial ovarian cancer cells by transactivation of the epidermal growth factor receptor. Endocr Relat Cancer 2010;17:929–40. doi: 10.1677/ERC-10-0107 20702723 10.1677/ERC-10-0107

[84] Al-Bataineh MM, van der MD, Schultz BD, Gehring R. Tumor necrosis factor alpha increases P-glycoprotein expression in a BME-UV in vitro model of mammary epithelial cells. BiopharmDrug Dispos 2010;31:506–15. 10.1002/bdd.731 PMC3034978 21104926

[85] Yu C, Kastin AJ, Tu H, Waters S, Pan W. TNF activates P-glycoprotein in cerebral microvascular endothelial cells. Cell Physiol Biochem 2007;20:853–8. doi: 10.1159/000110445 17982267 10.1159/000110445

[86] Bauer B, Hartz AMS, Miller DS. Tumor necrosis factor alpha and endothelin-1 increase P-glycoprotein expression and transport activity at the blood-brain barrier. Mol Pharmacol 2007;71:667–75. doi: 10.1124/mol.106.029512 17132686 10.1124/mol.106.029512

[87] Masereeuw R, Heemskerk S, Peters JGP, Louisse J, Sagar S, Russel FGM. Regulation of P-glycoprotein in renal proximal tubule epithelial cells by LPS and TNF-?? J Biomed Biotechnol 2010;2010 doi: 10.1155/2010/525180 20300455 10.1155/2010/525180 PMC2841251

[88] Yan B, Wang H, Rabbani ZN, Zhao Y, Li W, Yuan Y, et al Tumor necrosis factor-?? is a potent endogenous mutagen that promotes cellular transformation. Cancer Res 2006;66:11565–70. doi: 10.1158/0008-5472.CAN-06-2540 17178846 10.1158/0008-5472.CAN-06-2540

[89] Rajput S, Volk-Draper LD, Ran S. TLR4 is a novel determinant of the response to paclitaxel in breast cancer. Mol Cancer Ther 2013;12:1676–87. doi: 10.1158/1535-7163.MCT-12-1019 23720768 10.1158/1535-7163.MCT-12-1019 PMC3742631

[90] Ran S. The role of TLR4 in chemotherapy-driven metastasis. Cancer Res 2015;75:2405–10. doi: 10.1158/0008-5472.CAN-14-3525 25998620 10.1158/0008-5472.CAN-14-3525 PMC4470764

[91] Byrd CA, Bornmann W, Erdjument-Bromage H, Tempst P, Pavletich N, Rosen N, et al Heat shock protein 90 mediates macrophage activation by Taxol and bacterial lipopolysaccharide. Proc Natl Acad Sci U S A 1999;96:5645–50. 10318938 10.1073/pnas.96.10.5645 PMC21914

[92] Berkova N, Page M. Addition of hTNFα potentiates cytotoxicity of taxol in human ovarian cancer lines. Anticancer Res 1995;15:863–6. 7645972

[93] Zhang Y, Wang Y, Yuan J, Qin W, Liu F, Wang F, et al Toll-like receptor 4 ligation confers chemoresistance to docetaxel on PC-3 human prostate cancer cells. Cell Biol Toxicol 2012;28:269–77. doi: 10.1007/s10565-012-9221-2 22648782 10.1007/s10565-012-9221-2

[94] Uhlen M, Fagerberg L, Hallstrom BM, Lindskog C, Oksvold P, Mardinoglu A, et al Tissue-based map of the human proteome. Science (80-) 2015;347:1260419–1260419. doi: 10.1126/science.1260419 25613900 10.1126/science.1260419

[95] Kawasaki K, Gomi K, Kawai Y, Shiozaki M, Nishijima M. Molecular basis for lipopolysaccharide mimetic action of Taxol and flavolipin. J Endotoxin Res 2003;9:301–7. doi: 10.1179/096805103225002548 14577846 10.1179/096805103225002548

[96] Dowling JK, Mansell A. Toll-like receptors: the swiss army knife of immunity and vaccine development. Clin Transl Immunol 2016;5:e85 doi: 10.1038/cti.2016.22 27350884 10.1038/cti.2016.22 PMC4910119

[97] JOHNSON AG, GAINES S, LANDY M. Studies on the O antigen of Salmonella typhosa. V. Enhancement of antibody response to protein antigens by the purified lipopolysaccharide. J Exp Med 1956;103:225–46. 13286429 10.1084/jem.103.2.225 PMC2136584

[98] Baldridge JR, Cluff CW, Evans JT, Lacy MJ, Stephens JR, Brookshire VG, et al Immunostimulatory activity of aminoalkyl glucosaminide 4-phosphates (AGPs): induction of protective innate immune responses by RC-524 and RC-529. J Endotoxin Res 2002;8:453–8. doi: 10.1179/096805102125001064 12697089 10.1179/096805102125001064

[99] Moore A, McCarthy L, Mills KH. The adjuvant combination monophosphoryl lipid A and QS21 switches T cell responses induced with a soluble recombinant HIV protein from Th2 to Th1. Vaccine 1999;17:2517–27. 10418898 10.1016/s0264-410x(99)00062-6

