# Supplementary figures and images for: Inflammatory cytokine production in tumor cells upon chemotherapy drug exposure or upon selection for drug resistance
Source: PLoS One. 2017 Sep 15;12(9):e0183662. doi: 10.1371/journal.pone.0183662 (PMC5600395; doi:10.1371/journal.pone.0183662)

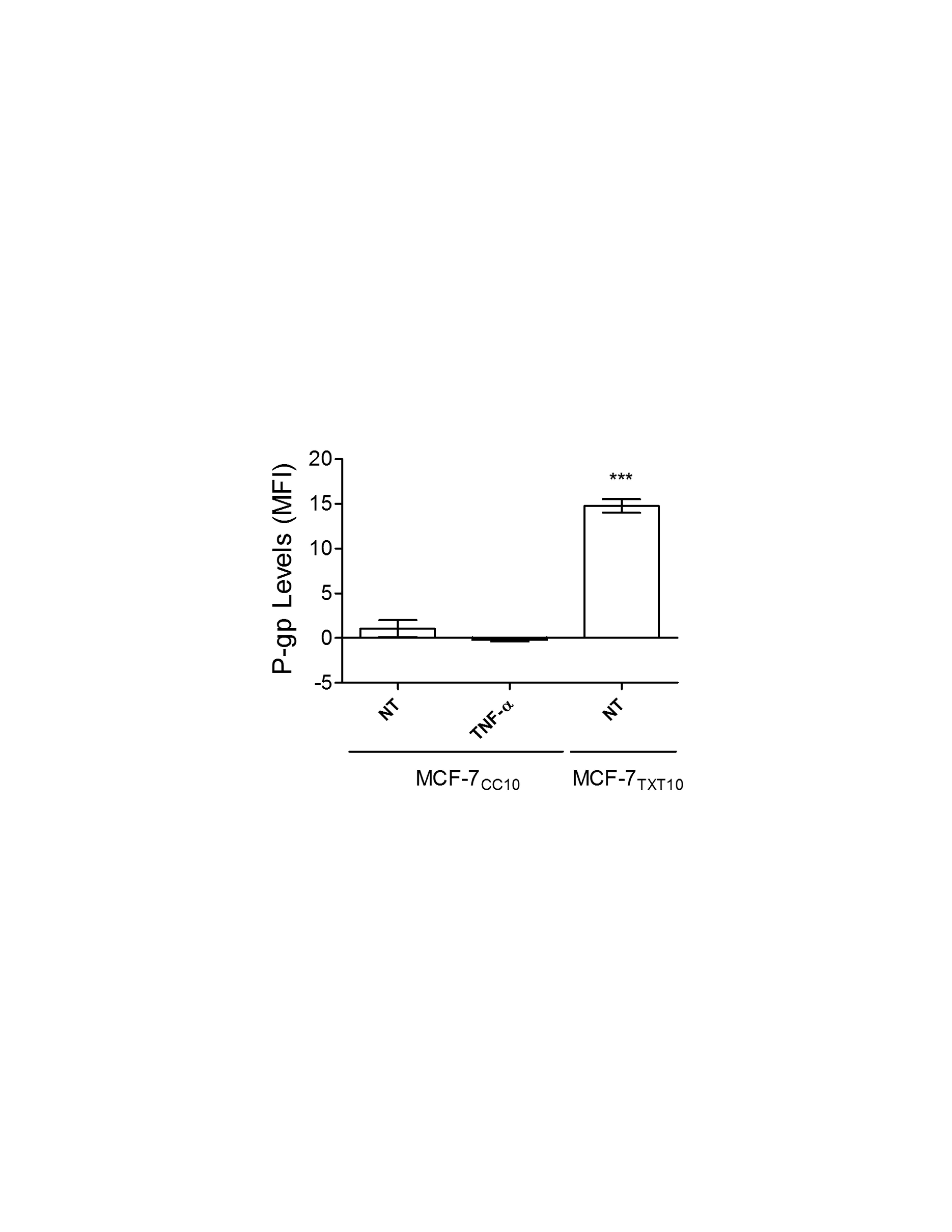

Supplement: S1 Fig — The medium of MCF-7CC10 cells was supplemented with 10 ng/ml TNF-α twice over 96 hours (second treatment at 48 hours), after which cell surface P-gp protein expression was assessed in treated and untreated cells by flow cytometry. This was then compared to P-gp expression in untreated MCF-7TXT10 cells using the same approach. An ANOVA with Tukey post-test was then used to assess the significance of differences in P-gp expression among the samples (***p = 0.0002). (TIF) [file pone.0183662.s001.tif]
