# Supplementary material for: Inflammatory cytokine production in tumor cells upon chemotherapy drug exposure or upon selection for drug resistance
Source: PLoS One. 2017 Sep 15;12(9):e0183662. doi: 10.1371/journal.pone.0183662 (PMC5600395; doi:10.1371/journal.pone.0183662)
Supplement: S1 File — (PDF) [file pone.0183662.s002.pdf]

RAW DATA FILES FOR

EDWARDSON ET AL.

Inflammatory Cytokine Production in Tumor Cells Upon Chemotherapy Drug Exposure or Upon Selection for Drug Resistance

PONE-D-17-07769

FIGURE 1

Panel A

	NT			2.5 nM Docetaxel		
	**A:Y1**	**A:Y2**	**A:Y3**	**B:Y1**	**B:Y2**	**B:Y3**
24h	0.113538	0.145884	0.112556	0.087251	0.128359	0.1081
48h	0.115496	0.166232	0.130045	1.109651	1.248917	1.182244
72h	0.285243	0.229331	0.226879	2.522443	2.076122	2.470708
96h	0.214771	0.24559	0.225553	3.29204	3.337343	2.953104

Panel B

	NT			2.5 nM Docetaxel		
	**A:Y1**	**A:Y2**	**A:Y3**	**B:Y1**	**B:Y2**	**B:Y3**
24h	0.213056	0.223397	0.212101	0.243376	0.247883	0.217154
48h	0.417918	0.511684	0.454801	1.75034	1.639714	2.0478
72h	0.852225	0.908543	0.869977	5.074019	5.390326	5.496852
96h	2.110896	1.988949	2.038824	9.660055	10.91504	10.77318

Panel C

	nM Docetaxel			
**NT**	**1.25**	**2.5**	**5**	**10**
0.126	2.323496	1.885323	1.758744	0.544212
0.17082	2.051868	1.512402	1.846681	0.572855
0.15887	2.219437	1.584118	1.868666	0.598044
0.05147	1.663898	1.81736	0.705269	0.574956
0.16297	1.677798	1.968242	0.794356	0.616025
0.07091	1.718234	2.142616	0.748391	0.517461
0.05592		2.356228		
0.05307		2.550179		
0.09209		2.46444		
0.1161		2.729738		
0.10425		2.785622		
0.08056		2.815714		
		2.685298		
		2.650424		
		2.77404		
		1.31924		
		1.425386		
		1.258585		
		0.750602		
		0.636875		
		0.761817		

Panel D

	nM Docetaxel			
**NT**	**1.25**	**2.5**	**5**	**10**
0.60948	5.95069	18.94217	27.3777	11.94902
0.6385	5.624236	18.0682	27.50985	10.76
0.55228	3.105399	4.438651	24.2262	6.380013
0.63064	3.20095	4.295962	27.1801	
0.59624	3.017705	4.60045	27.3246	
0.41599	5.748972	18.74869		
0.44502	5.443101	17.88706		
0.82027				
0.67252				

FIGURE 2

Panel A	Docetaxel nM				Paclitaxel nM			
NT NT	1.25 1.25	2.5 2.5	5 5	10 10	1.25 1.25	2.5 2.5	5 5	10 10
0.126	2.323496	1.885323	1.758744	0.544212	0.663852	1.157381	2.257469	0.427307
0.170823	2.051868	1.512402	1.846681	0.572855	0.627732	1.084831	2.034524	0.421732
0.15887	2.219437	1.584118	1.868666	0.598044	0.635205	1.126555	2.347768	0.385992
0.051466	1.663898	1.81736	0.705269	0.574956			0.732527	
0.162974	1.677798	1.968242	0.794356	0.616025			0.780562	
0.070908	1.718234	2.142616	0.748391	0.517461			0.638459	
0.055916		2.356228						
0.053073		2.550179						
0.092088		2.46444						
0.116097		2.729738						
0.10425		2.785622						
0.080557		2.815714						
		2.685298						
		2.650424						
		2.77404						
		1.31924						
		1.425386						
		1.258585						
		0.750602						
		0.636875						
		0.761817						

Panel B	Docetaxel nM				Paclitaxel nM			
NT	1.25	2.5	5	10	1.25	2.5	5	10
0.634342	30.28751	21.74215	19.8425	13.47882	2.322385	12.80064	21.41489	24.04128
0.614823	30.7121	22.27552	19.80046	13.269	2.557268	12.23623	21.71081	24.55925
0.460903	26.52423	30.5171	19.6332	13.46102	2.487504	12.75318	24.27309	15.91713
0.480917		30.74212						
0.455471		27.94868						
0.143505								
0.212285								
0.229268								

Panel C	Docetaxel nM				Paclitaxel nM			
NT	1.25	2.5	5	10	1.25	2.5	5	10
0.609478	5.95069	18.94217	27.3777	11.94902	0.869447	1.62692	6.797906	7.931286
0.6385	5.624236	18.0682	27.50985	10.76	0.931198	1.887095	6.418964	8.447143
0.552283	3.105399	4.438651	24.2262	6.380013	0.944498	1.632256	18.86743	9.182238
0.630635	3.20095	4.295962	27.1801				12.73777	3.672531

0.596237	3.017705	4.60045	27.3246				12.99656	3.927273
0.415993	5.748972	18.74869					9.048648	4.300045
0.445015	5.443101	17.88706					6.041207	
0.820269							6.178767	
0.672519								
0.578824								
0.515356								
0.575777								

FIGURE 3

Panel A

	TNF-alpha concentration in medium of MCF-7								
	**NT**			**2.5 nM Docetaxel**			**15 nM Docetaxel**		
	**A:Y1**	**A:Y2**	**A:Y3**	**B:Y1**	**B:Y2**	**B:Y3**	**C:Y1**	**C:Y2**	**C:Y3**
48 h	0.322246	0.1047144	0.072677	0.8255585	0.925993	1.004345	0.363112	0.429361	0.320221
72 h	0.222	0.2788887	0.31145	1.982784	2.247354	2.262005	0.408591	0.697569	0.568248

Panel B

	% Trypan-stained MCF-7								
	**No Treatment**			**2.5 nM Docetaxel**			**15 nM Docetaxel**		
	**A:Y1**	**A:Y2**	**A:Y3**	**B:Y1**	**B:Y2**	**B:Y3**	**C:Y1**	**C:Y2**	**C:Y3**
48 h	4.910331	5.574913	4.225352	8	6.410256	12.8	12.90323	16.66667	11.5942
72 h	5.7502	4.892214	4.190337	5.263158	8.333333	8.75	12.5	19.44444	9.259259

Panel C

**NT**	**2.5 nM Docetaxel**	**NT**	**2.5 nM Docetaxel**
0.116097	0.750602	0.0231595	0.212725
0.10425	0.636875	0.014296	0.152109
0.080557	0.761817	0.1222308	0.077198
media		lysate	

Panel D

**NT**	**2.5 nM Docetaxel**
0.764	2.81
0.558	7.95
0.691	16.8
0.817	4.52
	20.6

FIGURE 4

Panel A

NT NT	Docetaxel Docetaxel	LPS LPS
0.126	1.885323	6.704221
0.170823	1.512402	6.624016
0.15887	1.584118	6.58226
0.051466	1.81736	
0.162974	1.968242	
0.070908	2.142616	
0.055916	2.356228	
0.053073	2.550179	
0.092088	2.46444	
0.116097	2.685298	
0.10425	2.650424	
0.080557	2.77404	
	1.31924	
	1.425386	
	1.258585	
	0.7506022	
	0.6368746	
	0.761817	

Panel B

NT NT	Docetaxel Docetaxel	LPS LPS
0.609477	18.94217	0.7618468
0.6385	18.0682	0.7981253
0.552283	4.438651	0.5199906
0.630635	4.60045	0.5562691
0.596237	18.74869	
0.415993	17.88706	
0.445015		

Panel D

	nM Docetaxel			
NT NT	2.5 2.5	5 5	10 10	LPS LPS
8.992932	8.419748	9.239824	9.014973	8.643001
9.931528	8.985053	9.43871	8.690874	9.504001
8.241346	9.170523	9.694858	9.086047	7.956
9.089897	9.525413	8.563878		
8.990008	10.46637			
9.085901	10.90338			

Panel C	nM Docetaxel			
NT NT	2.5 2.5	5 5	10 10	LPS LPS
9.273304	11.34115	10.27908	11.20095	78.26811
8.823603	10.98123	10.33914	11.24546	81.12142
9.542653	13.53856	11.57783	11.13399	76.94978
9.36296	12.52252			
9.348104	11.62492			
9.10885	11.60303			

Panel E	nM Docetaxel			
NT NT	2.5 2.5	5 5	10 10	LPS LPS
0.2108705	0.283452	0.283452	0.260593	1.651347
0.2896642	0.384116	0.336985	0.269736	1.771146
0.1890563	0.302798	0.330475	0.208729	1.433388

Panel F	nM Docetaxel			
NT	2.5	5	10	LPS
0.6930309	0.735699	0.668125	0.472247	0.4792
0.5355238	0.71197	0.592149	0.428047	0.6279
0.5260399	0.645223	0.523449	0.423068	0.618
		0.583426		

FIGURE 5

Panel A	TNF-alpha concentration in MB-231 media				
NT	TXT	LPS	LPS-RS	TXT + LPS-RS	LPS + LPS-RS
0.598467	17.03487	3.866861	0.900544	24.47375	0.9139853
0.657573	15.71972	3.941303	0.771132	25.83559	0.8139535
0.732791	16.81154	6.793716	0.831703	25.84369	0.6323282

Panel B	TNF-alpha levels relative to untreated in MB-231 media				
NT	TXT	LPS	TAK-242	TXT + TAK-242	LPS + TAK-242
1.045333	18.52874	5.290668	0.877241	16.49361	1.824
0.954667	17.52828	5.535634	0.772414	16.99862	1.931034
1	8.907176	3.833853	0.950078	8.524961	1.121685

FIGURE 6

Panel A

NT	TXT	Marimastat	Marimastat + TXT	LPS	Marimastat + LPS
0.065389	2.380151	0.1052448	2.545491	7.948003	4.152964
0.074263	2.345277	0.0714606	2.920698	9.031589	4.778049
0.08267	2.452545	0.0616523	2.670819	7.099973	5.155903
0.119412	2.300062	0.1424211	1.791307	10.90422	5.053028
0.298297	2.239895	0.2056347	1.669449	10.63969	4.492737
0.255647					

Panel B

NT	TXT	Marimastat	Marimastat + TXT
0.852195	5.07384	0.1638837	3.048237
0.775214	5.390136	0.3380102	3.107235
0.843321	5.49666	0.1704391	3.089208

FIGURE 7

Panel A

MCF-7 CC10	TXT8	TXT9	TXT10	TXT11	TXT12
2.181673	0.053946	1.830803	17.73766	21.4299	55.48964
-0.238392	0.254337	5.946181	13.5049	13.98092	62.04313
2.002319	-0.092437	5.103512	22.41457	42.85769	50.03312
-1.02066					
0.0936					
0.3997					
-1.056726					
-0.31453					

Panel B

NT	Tar	H^3-TXT	H^3-TXT + Tar	NT	Tar	H^3-TXT	H^3-TXT + Tar
0.0445	0.008	14.9905	16.0905	0.124	0.0385	5.9875	12.2365
0.057	0.05	15.571	17.8775	0.06500001	0.047	6.762	13.304
0.049	0.037	16.094	15.9285	0.13	0.0572	6.5325	14.378

FIGURE 8

note: data points for the clonogenic curves are the 'Mean', which in this case refers to the mean number of colonies counted and then divided by the mean of the control cells (cells that received docetaxel at log concentration of -15)

Panel A

log [docetaxel]	MCF-7TXT10		MCF-7TXT10 + Tar (100nM)		MCF-7cc10		MCF-7cc10 + Tar (100nM)	
	Mean	SEM	Mean	SEM	Mean	SEM	Mean	SEM
-5	0.017964	0.524435	0.104651	0.184032				
-5.48	0.035928	0.305309	0.046512	0.288288				
-5.95	0.125749	0.190032	0.052326	0.246584				
-6.43	0.053892	0.336772	0.034884	0.46442				
-6.91	0.149701	0.168348	0.069767	0.184032				
-7.39	0.137725	0.20326	0.052326	0.296209				
-7.86	0.317365	0.137738	0.034884	0.307366				
-8.34	0.754491	0.096862	0.104651	0.246584				
-8.82	1.023952	0.078701	0.215116	0.14224				
-9.29	0.994012	0.08249	0.47093	0.090894				
-9.77	1.185629	0.065848	0.825581	0.087512				
-15	1	0.067891	1	0.084434				
-6.48					0.016722	0.358917*	0.0301	0.292369*
-6.95					0.046823	0.181903*	0.026756	0.33903*
-7.43					0.006689	0.675347*	0.036789	0.285824*
-7.91					0.043478	0.214933	0.023411	0.494315*
-8.39					0.133779	0.093922	0.153846	0.102043
-8.86					0.123746	0.134968	0.120401	0.134684
-9.34					0.287625	0.062125	0.297659	0.060287
-9.82					0.705686	0.064183	0.809365	0.051013
-10.29					0.849498	0.085196	0.816054	0.060819
-10.77					0.772575	0.074296	1.006689	0.057571
-11.25					0.889632	0.066649	1.177258	0.049192
-15					1	0.055654	1	0.051081

Panel B

Log[docetaxel]	A2780cc12 + TAR		A2780dxl12 + TAR		A2780cc12		A2780dxl12	
	Mean	SEM	Mean	SEM	Mean	SEM	Mean	SEM
-5.52	0.02551	0.239773	0.034856	0.24777	0.012672	0.272731	0.109137	0.246647
-6	0.041327	0.305563	0.044471	0.401342	0.026399	0.236144	0.192893	0.23255
-6.48	0.021429	0.387713	0.039663	0.37755	0.017951	0.287115	0.236041	0.1643
-6.95	0.018367	0.303762	0.027644	0.40671	0.024287	0.339229	0.332487	0.127448
-7.43	0.02551	0.272484	0.030048	0.32692	0.030623	0.40021	0.611675	0.110323
-7.91	0.037755	0.234222	0.030048	0.237924	0.030623	0.350442	0.949239	0.113952
-8.39	0.036735	0.199364	0.067308	0.181964	0.029567	0.301179	1.032995	0.110846
-8.86	0.115306	0.191799	0.126202	0.165093	0.263992	0.090422	1.005076	0.109116

-9.34	0.404082	0.121664	0.540865	0.079941	0.700106	0.065401	0.875635	0.096196
-9.82	0.860204	0.074396	0.84976	0.100283	1.089757	0.066986	1.068528	0.107529
-10.29	1.036735	0.106647	0.778846	0.082996	1.208025	0.081497	1.192893	0.092443
-15	1	0.082246	1	0.070694	1	0.057534	1	0.10704

FIGURE 9

(Panel A)

MCF-7 CC10	TXT7	TXT8	TXT9	TXT10	TXT11	TXT12
0.094824	0.029384	0.017331	0.84226	2.528026	0.280886	0.201999
0.109765	0.025897	-0.005777	0.978918	2.634201	0.20419	0.213653
0.124905	0.027391	-0.005478	0.918557	2.645686	0.193931	0.188254

(Panel C)

MCF-7 CC10	TXT7	TXT8	TXT9	TXT10	TXT11	TXT12
11.29996	31.93417	48.83867	81.86462	102.7976	33.03342	15.0359
9.792383	32.62696	49.54453	74.95169	95.19003	31.39389	14.44706
11.84771	33.56375	47.20848	79.0748	93.64388	35.74108	14.85664

(Panel E)

MCF-7 CC10	TXT7	TXT8	TXT9	TXT10	TXT11	TXT12
0.22707	0.470322	1.113426	6.6816	10.8837	1.098549	0.286578
0.252909	0.304326	1.141614	6.654978	10.62688	0.84564	0.20097
0.275094	0.267786	1.148139	6.828804	11.18124	0.97614	0.190008

(Panel B)

A2780 CC12	DXL10	DXL11	DXL12
0.341116	0.992879	3.032887	2.299098
0.407046	0.952004	3.18545	2.295106
0.372648	0.980351	3.130243	2.517758

(Panel D)

A2780 CC12	DXL10	DXL11	DXL12
9.805944	10.07681	9.295218	9.742544
10.39205	10.00777	8.305466	9.996146
10.62663	9.535435	9.543184	9.335724

(Panel F)

A2780 CC12	DXL10	DXL11	DXL12
0.018275	2.616266	1.566756	0.154197
0.054826	3.018663	2.018267	0.174757
0.099943	3.125059	1.880175	0.139977

FIGURE 10

MCF-7TXT10

NT	Marimastat	TXT	Marimastat + TXT	LPS	Marimastat + LPS
0.343812	0.243754	0.3470631	0.295641	2.585182	1.487322
0.358251	0.243903	0.3689741	0.297289	2.582885	1.433416
0.359813	0.185565	0.3858823	0.305803	2.799413	1.108661
0.360292	0.215526				
0.34817	0.23348				

FIGURE 11

Panel A

NT	TXT	LPS	NT	TXT	LPS
1	12.41692	33.49213	1	1.513043	9.932932
1	13.33832	32.69291	1	1.514578	9.924106
1	12.9274	33.44882	1	1.261722	10.75606

Panel B

NT	TXT	LPS	NT	TXT	LPS
1	27.50893	1.257038	1	0.895005	1.251036
1	30.58163	1.590821	1	1.050763	1.289561
1	30.13967	1.193984	1	1.178354	1.27794

Immunoblots for Figure 12
RAW DATA

TRIF Immunoblot Loading

1. $A2780_{CC12}$ Replicate 1
2. $A2780_{DXL12}$ Replicate 1
3. $A2780_{CC12}$ Replicate 2
4. $A2780_{DXL12}$ Replicate 2
5. $A2780_{CC12}$ Replicate 3
6. $A2780_{DXL12}$ Replicate 3
7. $MCF\text{-}7_{CC10}$ Replicate 1
8. $MCF\text{-}7_{TXT10}$ Replicate 1
9. $MCF\text{-}7_{CC10}$ Replicate 2
10. $MCF\text{-}7_{TXT10}$ Replicate 2
11. $MCF\text{-}7_{CC10}$ Replicate 3
12. $MCF\text{-}7_{TXT10}$ Replicate 3

TRIF Antibody 1:700

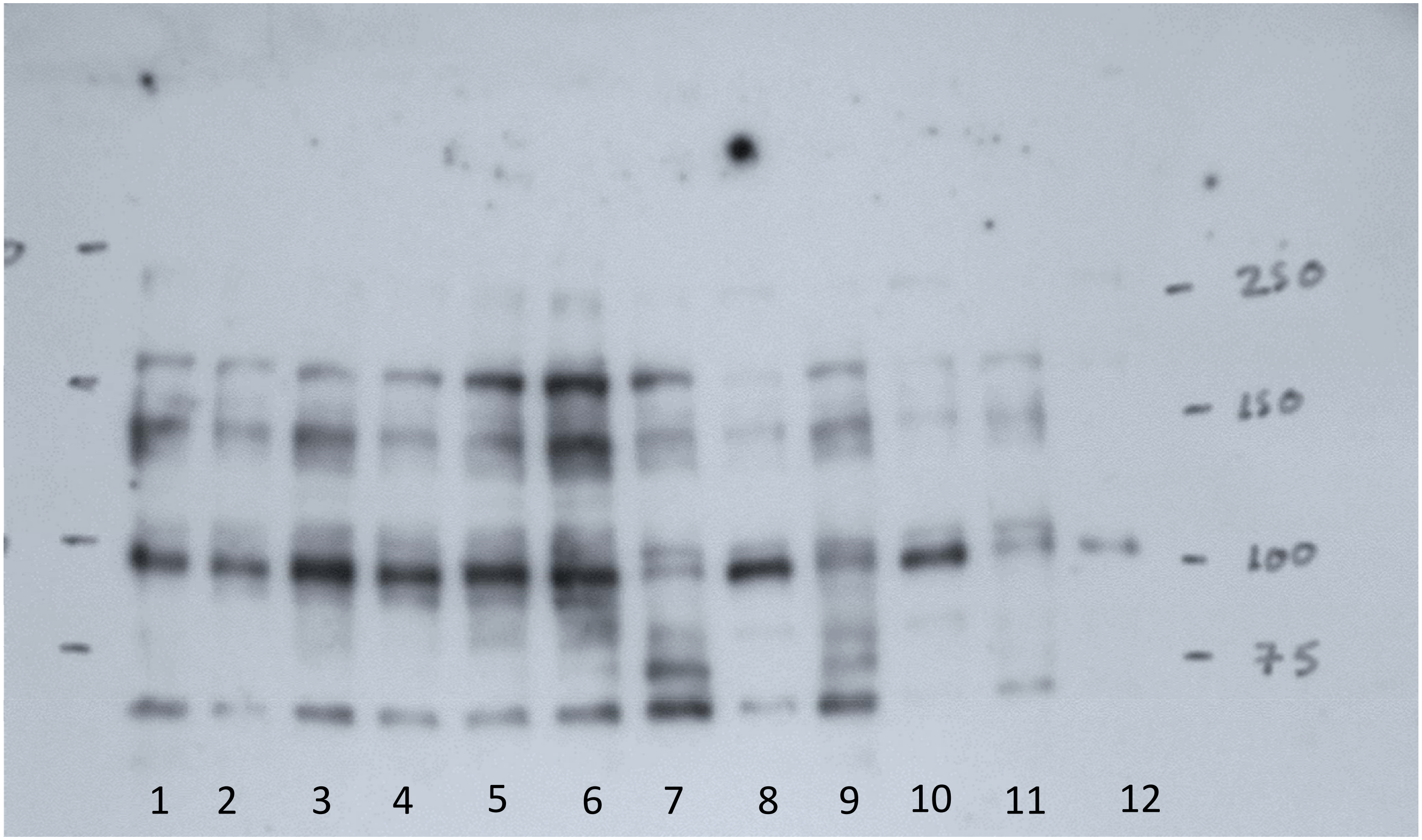

Gamma Tubulin Antibody 1:10,000 Loading Control for TRIF Immunoblot

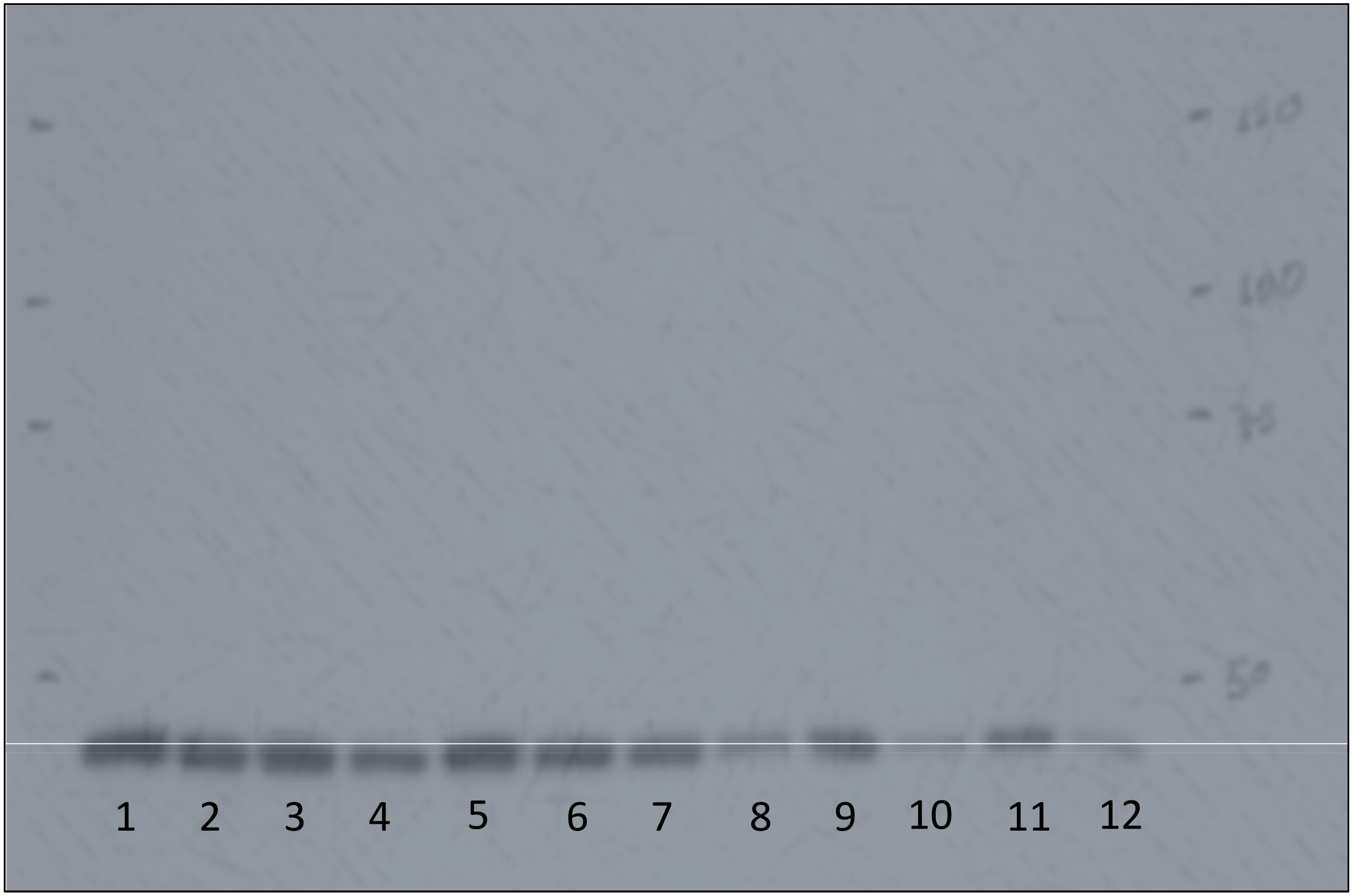

MyD88 Antibody 1:1000

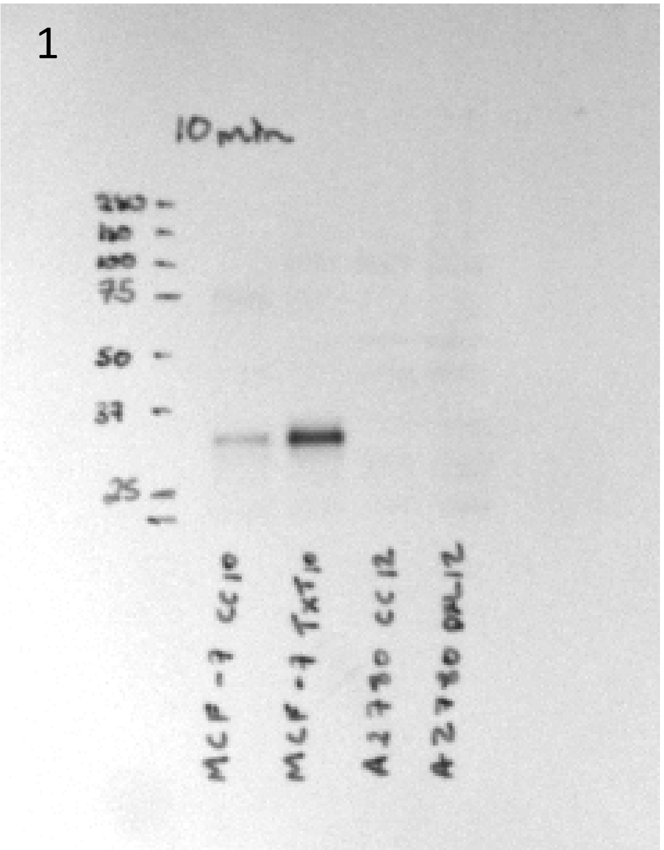

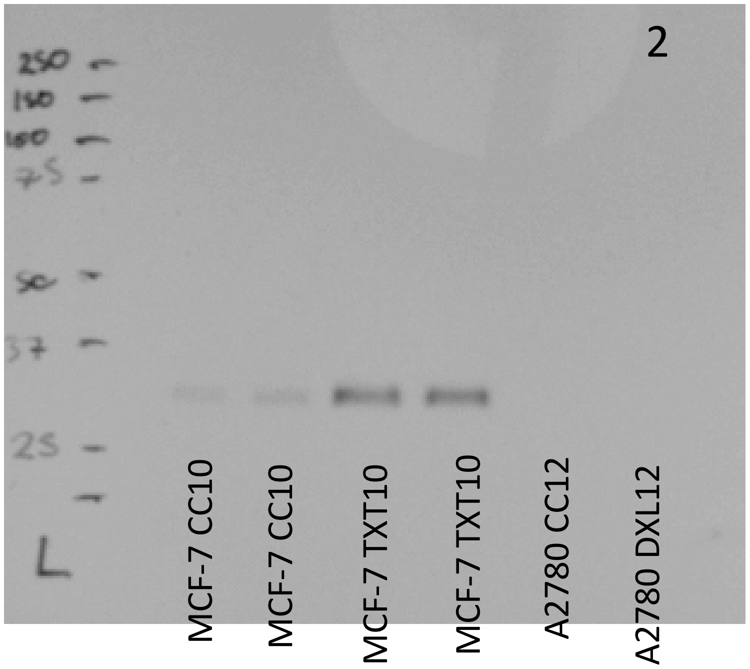

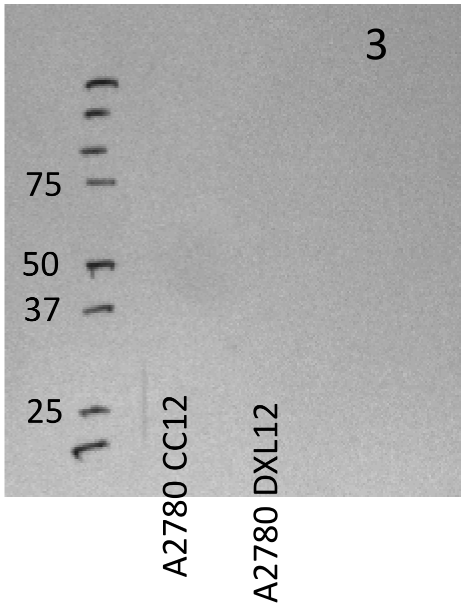

GAPDH Loading Controls 1:10000
For MyD88 Immunoblots

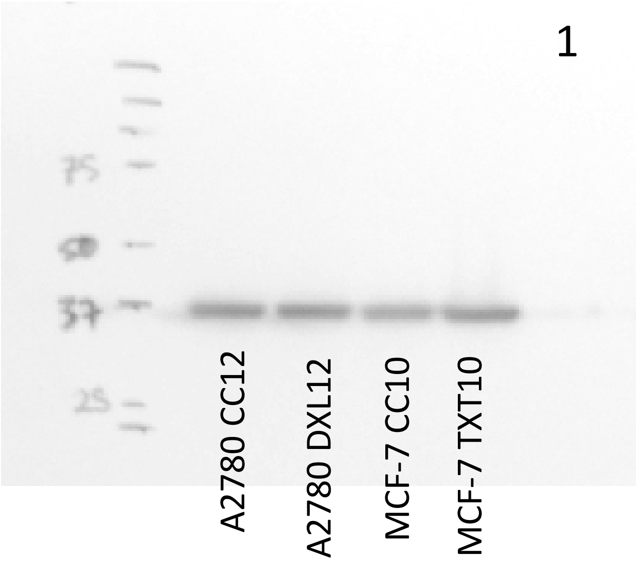

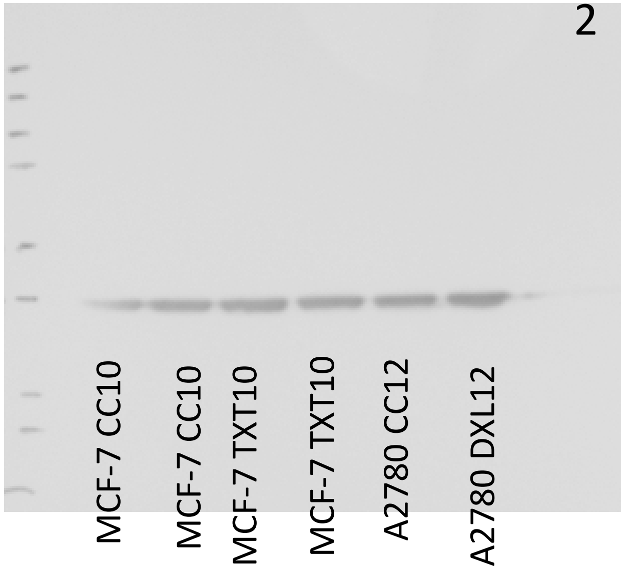

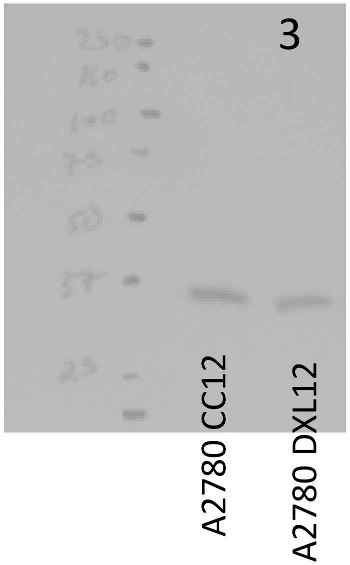

TLR4 Antibody: 1:250

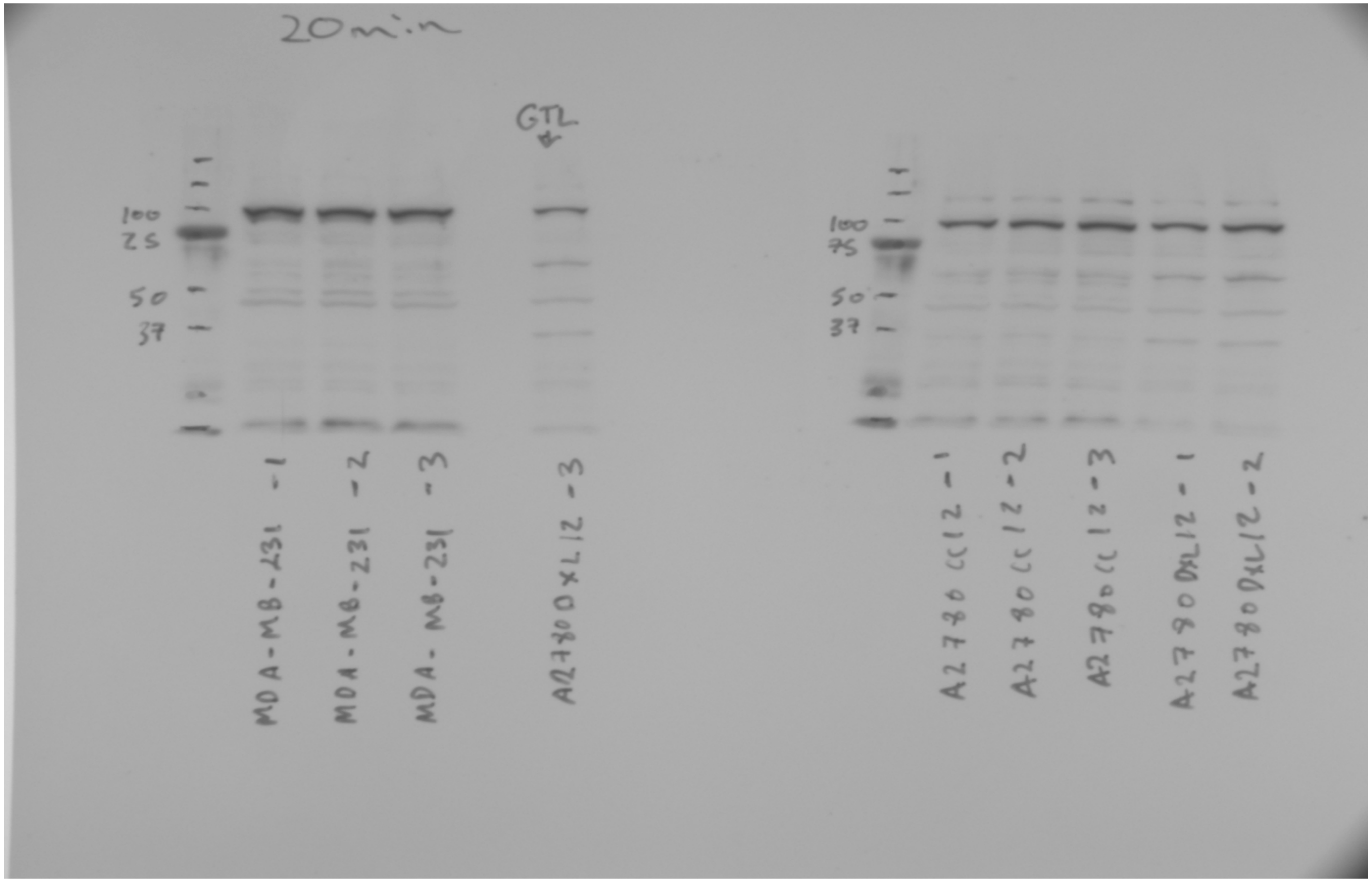

GAPDH Loading Control 1:10,000
For TLR4 Immunoblot

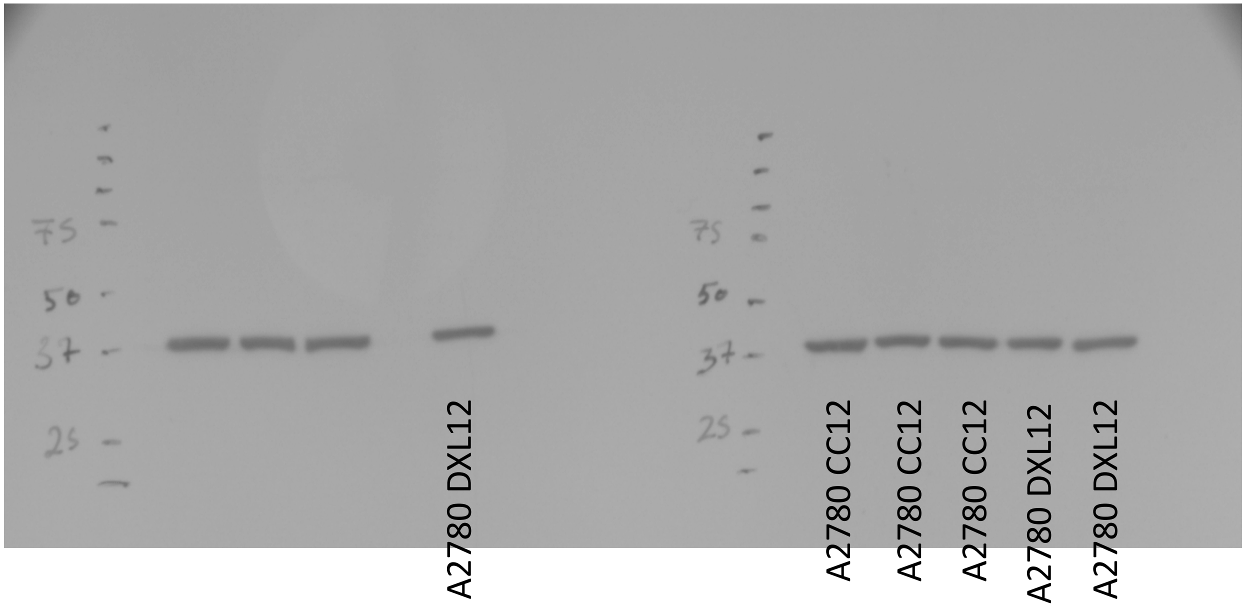

TLR4 Antibody 1:250

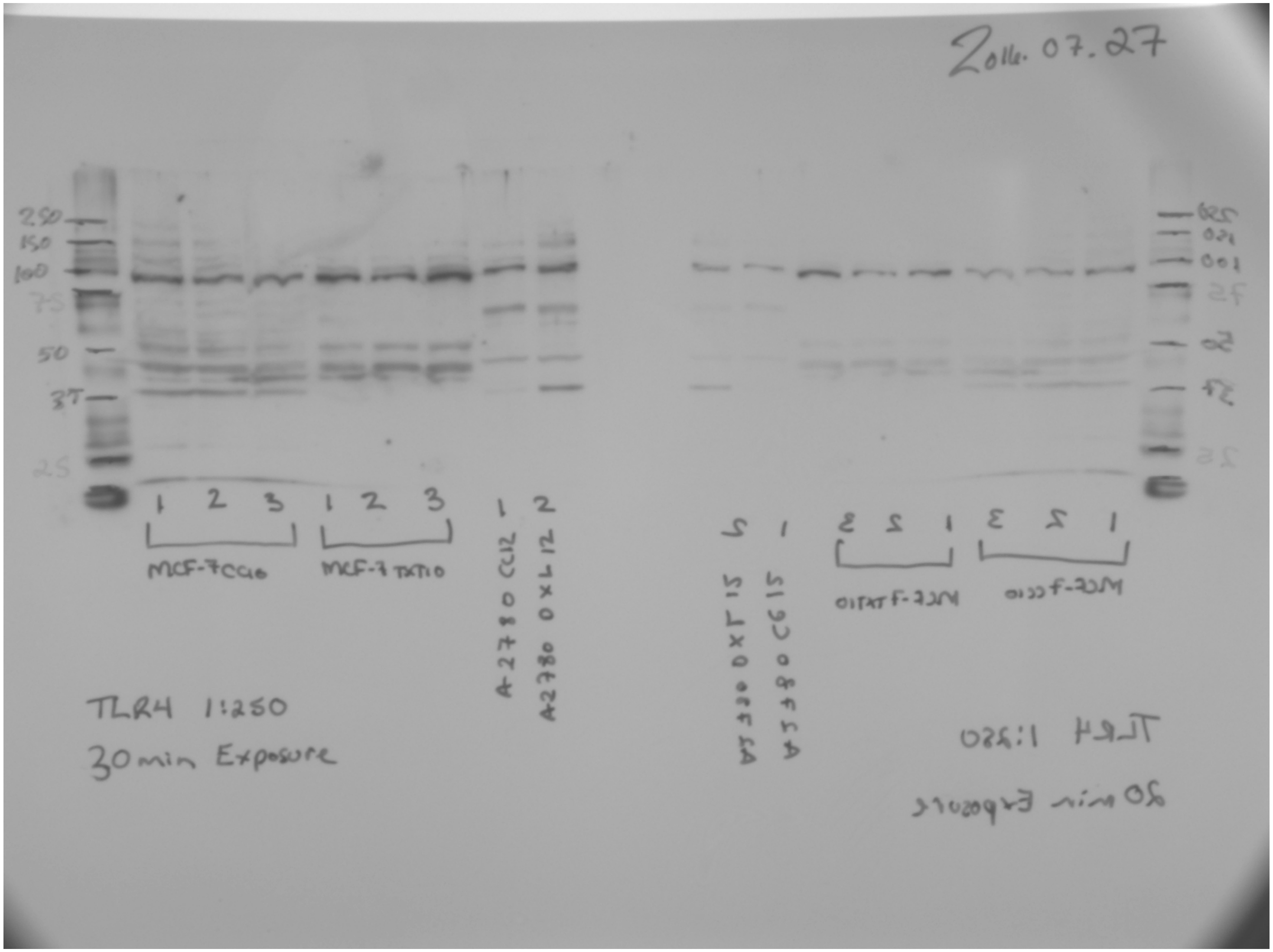

GAPDH Loading control 1:10,000
For Prior TLR4 Immunoblot

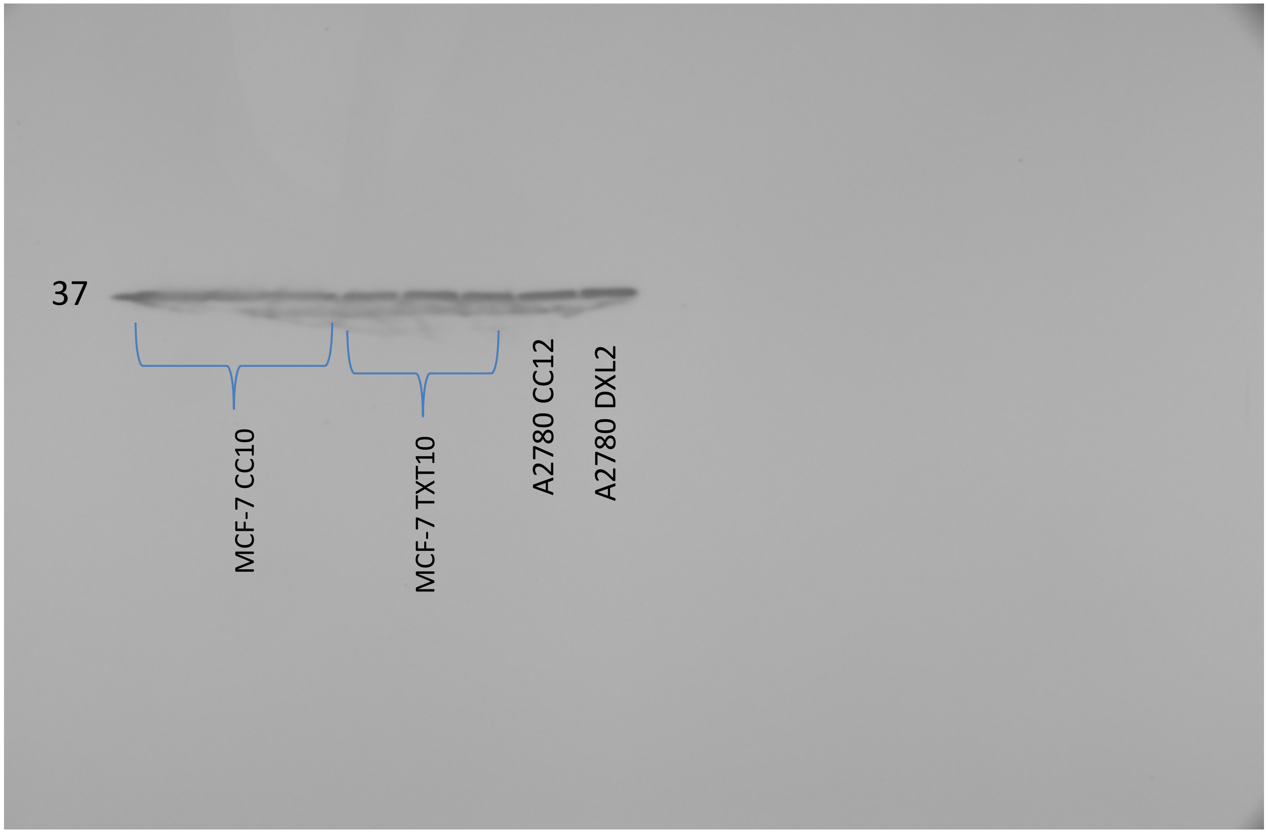

FIGURE 12

Panel C

	MCF-7 CC10			MCF-7 TXT10		
	A:Y1	A:Y2	A:Y3	B:Y1	B:Y2	B:Y3
TLR4	1.033333	0.884615	0.84	1.222222	0.83871	1.28125
MyD88	0.230769	0.181818	0.142857	0.8	0.5	0.521739

Panel E

	MCF-7 CC10	MCF-7 TXT10
	0.489796	0.848101
	0.433962	0.833333
TRIF	0.727273	0.764706

Panel D

	A2780 CC12			A2780 DXL12		
	A:Y1	A:Y2	A:Y3	B:Y1	B:Y2	B:Y3
TLR4	2.25	3.210526	3.045455	2.4	2.52381	1.952381
MyD88	0	0	0	0	0	0

Panel F

	A2780 CC12	A2780 DXL12
	0.897059	0.87931
	0.805556	0.836957
TRIF	0.669811	0.47541

FIGURE 13

Panel A

**MCF-7cc10**				MCF-7txt10			
**NT**	**TXT**	**Tariquida r**	**Tariquidar + TXT**	**NT**	**TXT**	**Tariquida r**	**Tariquidar + TXT**
1.020604	3.335888	0.847628	3.307618	0.9336385	1.436928	0.799914	1.963387
1.103977	3.612842	0.889315	2.89794	1.050343	1.425057	0.780892	2.031178
0.875419	3.932917	0.94873	3.48826	1.016018	1.222826	0.834096	2.202517

Panel B

A2780cc12				A2780dxl12			
**NT**	**TXT**	**Tariquida r**	**Tariquidar + TXT**	**NT**	**TXT**	**Tariquida r**	**Tariquidar + TXT**
0.982068	13.92257	0.985736	14.13653	0.9713228	0.895005	0.938714	2.00703
1.005604	12.24697	1.044422	12.16505	0.96716	1.050763	0.820074	1.93321
1.012328	12.23688	1.146205	11.64177	1.061517	1.178354	0.871485	2.048104

FIGURE 14

note: data points for the clonogenic curves are the values in the 'Mean' column, which in this case refers to the mean number of colonies counted and then divided by the mean of the control cells (cells that received docetaxel at log concentrationof -15)

Panel A

log [docetaxel]	MCF-7TXT10			MCF-7TXT10 + LPS (10ug/ml)			MCF-7cc10			MCF-7cc10 + LPS (10ug/ml)		
	Mean	SEM	N	Mean	SEM	N	Mean	SEM	N	Mean	SEM	N
-5	0.049587	0.331724*	12	0.028986	0.286971*	12						
-5.48	0.024793	0.464867*	12	0.007246	0.676278*	12						
-5.95	0.053719	0.220517*	12	0.028986	0.42968*	12						
-6.43	0.090909	0.239423*	12	0.036232	0.360666*	12						
-6.91	0.202479	0.127318	12	0.083333	0.223754	12						
-7.39	0.454545	0.134889	12	0.155797	0.152996	12						
-7.86	0.714876	0.099956	12	0.416667	0.10472	12						
-8.34	1.243802	0.08439	12	0.597826	0.07281	12						
-8.82	1.057851	0.103519	12	0.702899	0.081027	12						
-9.29	1.289256	0.087881	12	1.057971	0.088057	12						
-9.77	1.132231	0.07188	12	1.021739	0.064537	12						
-15	1	0.08922	12	1	0.074925	12						
-6.48							0.029412	0.570453*	12	0.015	0.719802*	12
-6.95							0.022059	0.529115*	12	0.015	0.719802*	12
-7.43							0.051471	0.341609*	12	0.02	0.566697*	12
-7.91							0.095588	0.341223*	12	0.045	0.295193*	12
-8.39							0.154412	0.228867	12	0.025	0.466379*	12
-8.86							0.323529	0.163613	12	0.155	0.148886	12
-9.34							0.448529	0.160252	12	0.185	0.121861	12
-9.82							0.698529	0.157328	12	0.385	0.105963	12
-10.29							1.117647	0.10509	12	0.625	0.093556	12
-10.77							0.698529	0.118806	12	0.86	0.08437	12
-11.25							1.147059	0.107417	12	0.96	0.081807	12
-15							1	0.120291	12	1	0.076989	12

Panel B

log [docetaxel] log [docetaxel]	A2780 $_{DXL12}$ A2780 $_{DXL12}$			A2780 $_{DXL12}$ + LPS A2780 $_{DXL12}$ + LPS			A2780 $_{CC12}$ A2780 $_{CC12}$			A2780 $_{CC12}$ + LPS A2780 $_{CC12}$ + LPS		
	Mean Mean	SEM SEM	N N	Mean Mean	SEM SEM	N N	Mean Mean	SEM SEM	N N	Mean Mean	SEM SEM	N N
-5	0		12	0		12						
-5.48	0.012821	0.718312*	12	0		12						
-5.95	0		12	0		12						
-6.43	0.012821	0.718312*	12	0.03211	0.332089*	12						
-6.91	0.141026	0.145281	12	0.059633	0.267	12						
-7.39	0.482906	0.115641	12	0.422018	0.077862	12						
-7.86	0.700855	0.082771	12	0.490826	0.059803	12						
-8.34	0.846154	0.052356	12	0.724771	0.080545	12						
-8.82	0.833333	0.0438	12	0.825688	0.067765	12						
-9.29	1.089744	0.066449	12	0.830275	0.071976	12						
-9.77	1.141026	0.043191	12	0.912844	0.069377	12						
-15	1	0.051324	12	1	0.070973	12						
-5.48							0		12	0		12
-5.96							0		12	0		12
-6.44							0		12	0		12
-6.91							0		12	0		12
-7.39							0		12	0		12
-7.87							0		12	0		12
-8.34							0		12	0		12
-8.82							0.442953	0.061858	12	0.238095	0.08237	12
-9.3							0.671141	0.051374	12	0.866667	0.04393	12
-9.78							0.852349	0.04904	12	1.257143	0.065782	12
-10.25							0.885906	0.07257	12	1.047619	0.05004	12
-15							1	0.055695	12	1	0.066019	12

S1 FIGURE

S1

NT	TNF- α	NT
2.582826	-0.386574	14.03643
-0.683493	-0.372971	16.23155
1.267765	0.111289	13.99746
MCF-7cc10		MCF-7txt10
